# Bond Length as a Unified Descriptor for Stable Iodine Battery

**DOI:** 10.1002/anie.9885697

**Published:** 2026-07-02

**Authors:** Mengzi Geng, Yanyan Wang, Fanbin Zeng, Yao Liu, Haijin Ni, Bolong Hong, Wei Xia, Songbai Han, Yusheng Zhao, Biao Zhang

**Affiliations:** ^1^ Department of Applied Physics and Research Institute for Advanced Manufacturing The Hong Kong Polytechnic University Kowloon Hong Kong China; ^2^ Eastern Institute for Advanced Study Eastern Institute of Technology Ningbo Ningbo China; ^3^ Shenzhen Key Laboratory of Solid‐State Batteries Southern University of Science and Technology Shenzhen China

**Keywords:** bond length, cathode design, electrolyte formulation, ligand, Li−iodine batteries

## Abstract

Dissolution of active materials in the electrolyte and their subsequent shuttling are common challenges for realizing stable electrodes in rechargeable batteries. These issues become particularly pronounced in cathodes with high solubility, including high‐energy iodine electrodes. The interaction strength of iodine with the host electrode and electrolyte is critical for determining electrochemical stability, yet there is a lack of an appropriate parameter to quantify it. Our findings reveal that, as a weak Lewis acid, iodine's interaction strength is highly influenced by the nucleophilicity of surrounding ligands. We propose the I−I bond length, which can be conveniently probed through Raman tests, as a unified descriptor to predict the iodine electrode stability. The asset of this descriptor is demonstrated in (i) rational design of complex electrodes to enhance the binding strength between iodine and host and (ii) efficient screening of electrolyte solvents to minimize the shuttle. The collective effects enable stable cycling of Li−I_2_ batteries under the challenging current rate of 0.1 C for over 4000 h. Overall, the unified descriptor provides a powerful means to expedite electrode and electrolyte design for overcoming active material dissolution challenges.

## Graphical abstract

Charge transfer induced by the interactions between iodine and ligands leads to the elongation of the I–I bond. This resulting bond length correlates directly with coordination strength, serving as a unified descriptor for the rational design of both cathode hosts and electrolyte components.

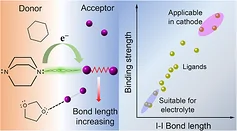

## 1 Introduction

Sustainable and high‐energy rechargeable batteries are essential to the electrification of our society [1, 2, 3]. The iodine cathode has garnered considerable attention due to its high energy density and wide availability in seawater. Extensive research, particularly on aqueous Zn−I_2_ batteries [4, 5, 6, 7], has been stimulated recently. Dissolution of iodine in the electrolyte is among the major challenging issues afflicting the practical application [8, 9, 10, 11]. Iodine has high solubility in most solvents, resulting in the deterioration of electrodes that leads to fast capacity degradation [12, 13, 14]. Furthermore, the polyiodides generated, such as triiodide, can diffuse toward the anode and be reduced, which may poison the anode and lead to a rapid self‐discharge rate [15]. These issues become even more pronounced in Li−I_2_ batteries, which hold much higher energy densities than their Zn counterparts, because of aggravated dissolution of iodine in organic electrolytes consisting of highly polar solvents [16, 17, 18].

The reduction of iodine produces iodide (I^–^) in the electrolyte, which further combines with I_2_ to generate triiodide (I_3_^–^). Efforts have been focused on trapping these polyiodides to prevent their migration to the anode. This can be achieved by inserting an additional blocking layer between the separator and cathode, capturing the dissolved species through either physical or chemical adsorption [19, 20]. Functional electrolyte additives have also been developed for immobilizing polyiodide via forming precipitate complexes [21, 22, 23]. Along with these, stabilizing pristine iodine through host electrode design can prevent the initial generation of these species. Encapsulation of iodine into activated carbon (AC) has become almost a standard protocol in iodine electrode preparation. Nevertheless, the weak Van der Waals forces between iodine and AC cannot effectively inhibit the iodine dissolution [24, 25, 26, 27]. Instead, intermolecular interactions through complexation of iodine with several organic hosts have demonstrated great capability in stabilizing iodine [28, 29]. This is well exemplified by recent works on utilizing amine‐containing organic materials to anchor iodine through the formation of N···I halogen bond [30, 31]. Weakening the iodine affinity with electrolytes is equally important. The wide variety of solvent candidates in organic electrolyte systems offers an opportunity to screen appropriate solvents. Quantifying the interaction strength of iodine with the host and electrolyte will facilitate the rapid screening of potential candidates but remains challenging.

Herein, we leverage coordination chemistry to guide the design of stable iodine batteries. Specifically, both the host electrode and the electrolyte solvent act as ligands, which bind to the Lewis acidic iodine molecules through the formation of charge‐transfer complexes with different coordination strengths. Ligands with high nucleophilicity that provide strong anchoring for iodine are therefore suitable as host materials, while solvents with low nucleophilicity are preferred to serve as electrolyte components to reduce competition with iodine‐complexed cathodes. We demonstrate that the coordination strength between the ligands and iodine is manifested in the I−I bond length, making it an effective descriptor of the interaction strength between iodine and the ligands. Encouragingly, Raman spectroscopy offers a rapid and convenient method to characterize I−I bond length, greatly facilitating the rational design of electrodes and electrolytes.

## 2 Results and Discussion

### 2.1 I−I Bond Length as a Unified Descriptor

**FIGURE 1:**
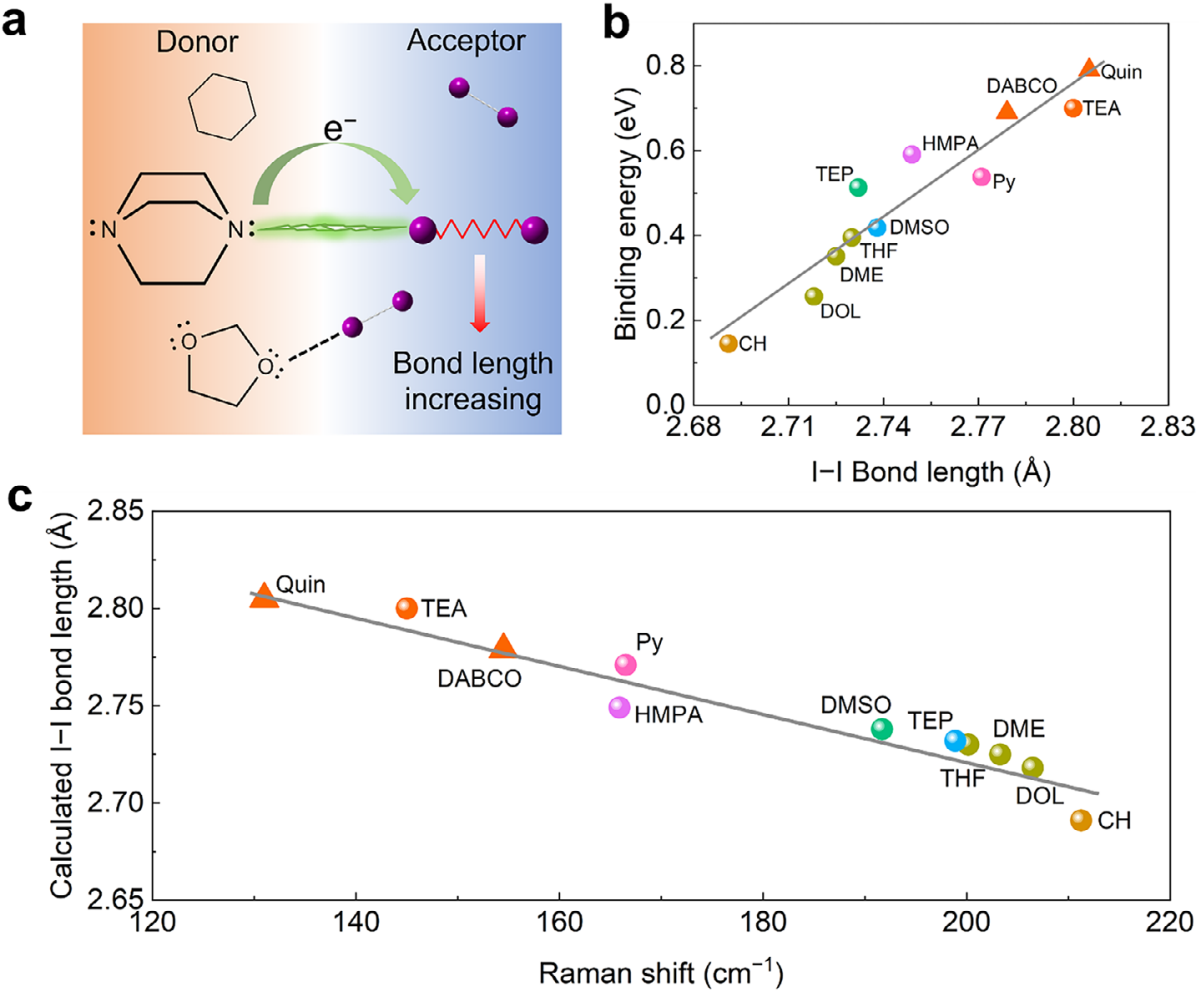
I−I bond length as a descriptor for iodine‐ligand interaction. (a) Illustration of the interaction between iodine and ligand; (b) Correlation between iodine‐ligand interaction strength and I−I bond length; (c) The reflectance of I−I bond length on the Raman shifts in iodine‐ligand complexes.

The large polarizability of the heavy iodine atom leads to the formation of a σ‐hole, i.e., an electron‐depleted area along the extension of the covalent bond, which acts as an electron‐accepting site. This is verified in the electrostatic potential (ESP) mapping through theoretical calculation (Figure S1), revealing a positively charged area at both ends of I−I bond. The formation of the σ‐hole is associated with the lowest unoccupied molecular orbital (LUMO) of iodine (Figure S2), serving as the preliminary acceptor orbital for the electrons donated by the ligands. As the highest occupied molecular orbital (HOMO) energy of ligands (which usually donates electrons during the reaction) increases and approaches the iodine LUMO energy, electron transfer from the ligand to iodine takes place (Figure 1a, Figure S3, and Supplementary Note 1). The electron injection into iodine elevates its LUMO energy level, thereby widening its HOMO‐LUMO gap. Consequently, the electron excitation of π* → σ* will require higher energy.

To verify the above assumption, we adopted UV‐Vis spectroscopy to explore the interaction of iodine with various solvents, including cyclohexane (CH), 1,2‐dimethoxyethane (DME), 1,3‐dioxolane (DOL), tetrahydrofuran (THF), triethyl phosphate (TEP), dimethyl sulfoxide (DMSO), hexamethylphosphoramide (HMPA), pyridine (Py), and triethylamine (TEA). As shown in Figures S4 and S5, with the variation of the HOMO level of ligands, iodine in the different ligand solvents exhibits pronounced absorption shifts, ranging from 520 nm in the weak ligand of CH to 400.7 nm in TEA. However, the HOMO energy level fails to adequately reflect the interaction strength between iodine and these ligands (Figure S6), which is ascribed to the fact that the HOMO describes the electron‐donating tendency of the entire molecule rather than the localized reactive site [32]. Therefore, a specific parameter that directly correlates with the interaction characteristics is indispensable for predicting the binding strength between iodine and various ligands.

Considering the LUMO of iodine corresponds to its σ* (antibonding) orbital, population of this orbital with additional electrons will reduce the I−I bond order, leading to an increase in I−I bond length in the iodine‐ligand complex, as illustrated in Figure 1a. This relationship is confirmed by the linearly increased I−I bond length with the number of electrons transferred from ligand solvents (Figure S7, Figure S8, and Table S1). On this basis, the I−I bond length, as a crucial parameter in iodine complexes, is proposed as an indicator of the interaction strength between iodine and its coordinating ligands.

To examine the hypothesis, DFT calculations were performed to examine the binding energy between iodine and different ligands. It is then correlated with the calculated I−I bond length obtained after electron injection, as summarized in Figure 1b. The observation substantiates the capability of I−I bond length in reflecting the binding strength, where elongation of the bond suggests increased affinity of the ligand for iodine. The conclusion is further supported by the experimental results on the excitation energy detected in UV‐Vis, which correlates well with the I−I bond length (Figure S9).

The distance between atoms in the diatomic molecules can be characterized by the Raman vibration mode based on Badger's rule and the harmonic oscillator model, which establishes connections between bond length *r*, force constant *k*, and vibration frequency *v* through the following equations [33, 34, 35]:

(1) $$r={\left(\frac{c_{0}}{k}\right)}^{\beta }+d_{0}$$

(2) $$k=\mu {\left(2\pi v\right)}^{2}\;$$

These lead to an overall equation as:

(3) $$r={\left(\frac{c_{0}}{4\pi^{2}\mu }\right)}^{\beta }{\left(v\right)}^{-2\beta }+d_{0}\;\;$$

where *c_0_* and *d_0_* are undetermined constants, and *μ* is the reduced mass associated with the vibrational atoms. The exponent *β*, with an original value of 1/3, can be optimized according to specific system [33, 35]. These relationships suggest the bond length can be quantified by the stretching frequency. Therefore, Raman shifts were used to determine the I−I bond length in iodine complexes and to infer their complexation strength (Figure S10). Figure 1c shows the relationship between the bond length derived from DFT calculation and the Raman shifts. A linear dependence was found, consistent with previous report [33], demonstrating that Raman shifts provide a reliable representation of the I−I bond length.

**FIGURE 2:**
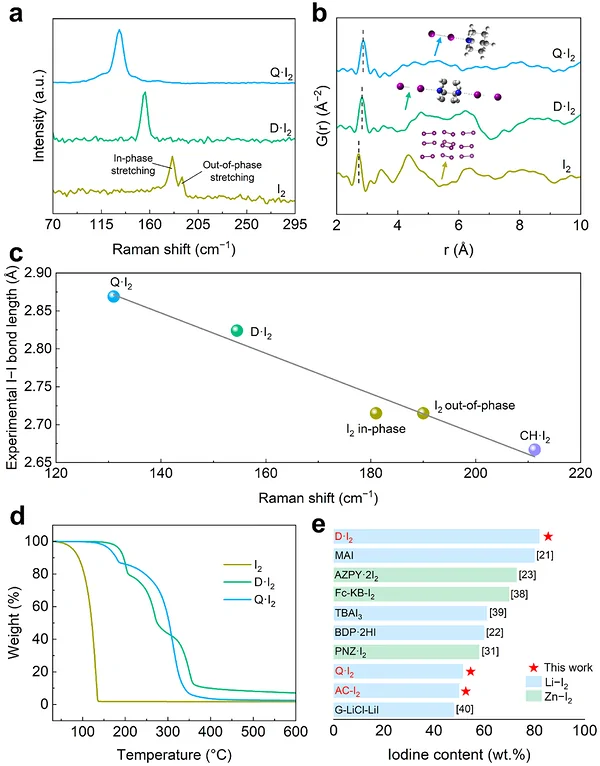
Host electrode design. (a) Raman spectra of I_2_, D·I_2_ and Q·I_2_; (b) PDF curves of I_2_, D·I_2_ and Q·I_2_; (c) The correlation between I−I bond length determined by PDF and Raman shift. The bond length data of CH·I_2_ was adopted from ref [37].; (d) TGA curves of I_2_, D·I_2_ and Q·I_2_; (e) Comparison of iodine content with reported works [21, 22, 23, 31, 38, 39, 40].

The descriptor of I−I bond length can be used not only to investigate the interaction of iodine with solvent ligands, but also to determine suitable host electrodes. Strong binding of iodine to the host can significantly suppress its dissolution into electrolytes. To investigate this effect, we prepared two model electrodes by incorporating iodine into two different host materials, i.e., Quinuclidine (Quin) and 1,4‐diazabicyclo[2.2.2]octane (DABCO). They possess high nucleophilicity, which is supposed to effectively confine iodine through their interactions with σ‐holes. The iodine complexes of Quin·I_2_ (Q·I_2_) and DABCO·I_2_ (D·I_2_) were synthesized by simply mixing the precursors in the ethanol solution. Both DABCO and Quin act as electron donors when interacting with iodine. Similar to the case with solvent ligands, this electron transfer leads to elongation of the I−I bond and the red shift of Raman peak. This effect is confirmed by the Raman results in Figure 2a, where the I−I vibrational modes exhibit low Raman shifts of 154.5 and 131.0 cm^−^^1^ for D·I_2_ and Q·I_2_, respectively. These values are lower than the Raman shifts observed for iodine in most solvents, indicating stronger interactions between iodine and the two hosts, which is further verified by the high binding energies of 0.69 and 0.79 eV, respectively (Figure 1b and Table S1). Furthermore, we calculated the I−I bond lengths in D·I_2_ and Q·I_2_. Notably, the relationship between Raman shift and I−I bond length is consistent with the correlation previously established for solvent ligands, as shown by the triangular data points in Figure 1c. This demonstrates the general applicability of using I−I bond length as a descriptor for assessing the interaction strength of iodine toward both solvents and host materials, with Raman spectroscopy providing a convenient method for efficient screening.

We further adopt x‐ray total scattering to experimentally probe the I−I bond length through analyzing atomic pair distribution function (PDF). PDF analysis in Figure 2b shows an I−I bond length of 2.715 Å in iodine crystals, in agreement with a previous report [36]. This value is higher than the reported bond length of 2.67 Å for CH·I_2_ [37], which is attributed to the stronger intermolecular interaction within iodine crystals, contrasted to the negligible interaction between iodine and CH. In comparison, the I−I bond lengths in D·I_2_ and Q·I_2_ are 2.824 and 2.869 Å, respectively, both significantly longer than that observed in iodine crystals. This increase is attributed to greater electron injection from the host electrode ligand. Furthermore, the I−I bond length exhibits an inversely proportional relationship with the Raman shifts, as shown in Figure 2c. The strong agreement between computational and experimental results confirms the reliability of Raman spectroscopy for probing the I−I bond length, which reflects the interaction strength between iodine and the ligands.

### 2.2 Host Cathode and Electrolyte Component Design

Considering their strong binding with iodine, D·I_2_ and Q·I_2_ were further explored as cathodes in Li−I_2_ batteries. The Raman spectra showed that, in addition to the I−I stretching vibration, a new low‐frequency peak appeared. As shown in Figure S11, the peaks at 64 cm^−1^ in D·I_2_ and 49 cm^−1^ in Q·I_2_ were ascribed to intermolecular N···I vibrations [41], suggesting the formation of a dative covalent bond. The formation of the covalent bond between iodine and the ligand endows iodine with higher thermal stability. As shown in Figure 2d, significant weight loss is observed even at room temperature for iodine due to its volatility, whereas the onset temperature of weight loss increases to 150°C for Q·I_2_ and 170°C for D·I_2_. This endows iodine complexes with enhanced processability. The iodine content in the two complexes was then measured by inductively coupled plasma‐mass spectrometry (ICP‐MS) (Figure 2e and Figure S12). Due to the bidentate structure of DABCO, D·I_2_ achieves an iodine content of around 81%, approaching the theoretical value expected for the stoichiometric compound of DABCO and I_2_ with a molar ratio of 1:2. Moreover, benefiting from the bidentate property of both DABCO and I_2_, D·I_2_ may form chain‐like geometry, supported by the rod‐like morphology (Figure S13). By contrast, monodentate Quin shows lower binding capacity toward iodine, as indicated by the iodine content of only 51% in Q·I_2_. D·I_2_ exhibits higher iodine content than most reported iodine complex cathodes and traditional AC‐I_2_ compounds (Figure 2e and Figure S14), beneficial for improving the energy density of batteries. Combining with the low cost of DABCO, D·I_2_ was selected as the active materials for further analysis. It is worth mentioning that the D·I_2_ reported here significantly differs from widely reported ammonium iodides, such as DABCO·HI (D·HI) [42], where no distinct I−I or N···I vibrational feature is detected (Figure S15 and Supplementary Note 2). In comparison, the stretching and bending modes of N−H are found in attenuated total reflectance Fourier transform infrared spectra (ATR‐FTIR) of D·HI (Figure S16). The covalently bonded D·I_2_ is speculated to achieve a higher iodine conversion ratio and enhanced stability due to its ability to bind with iodine oxidation products.

**FIGURE 3:**
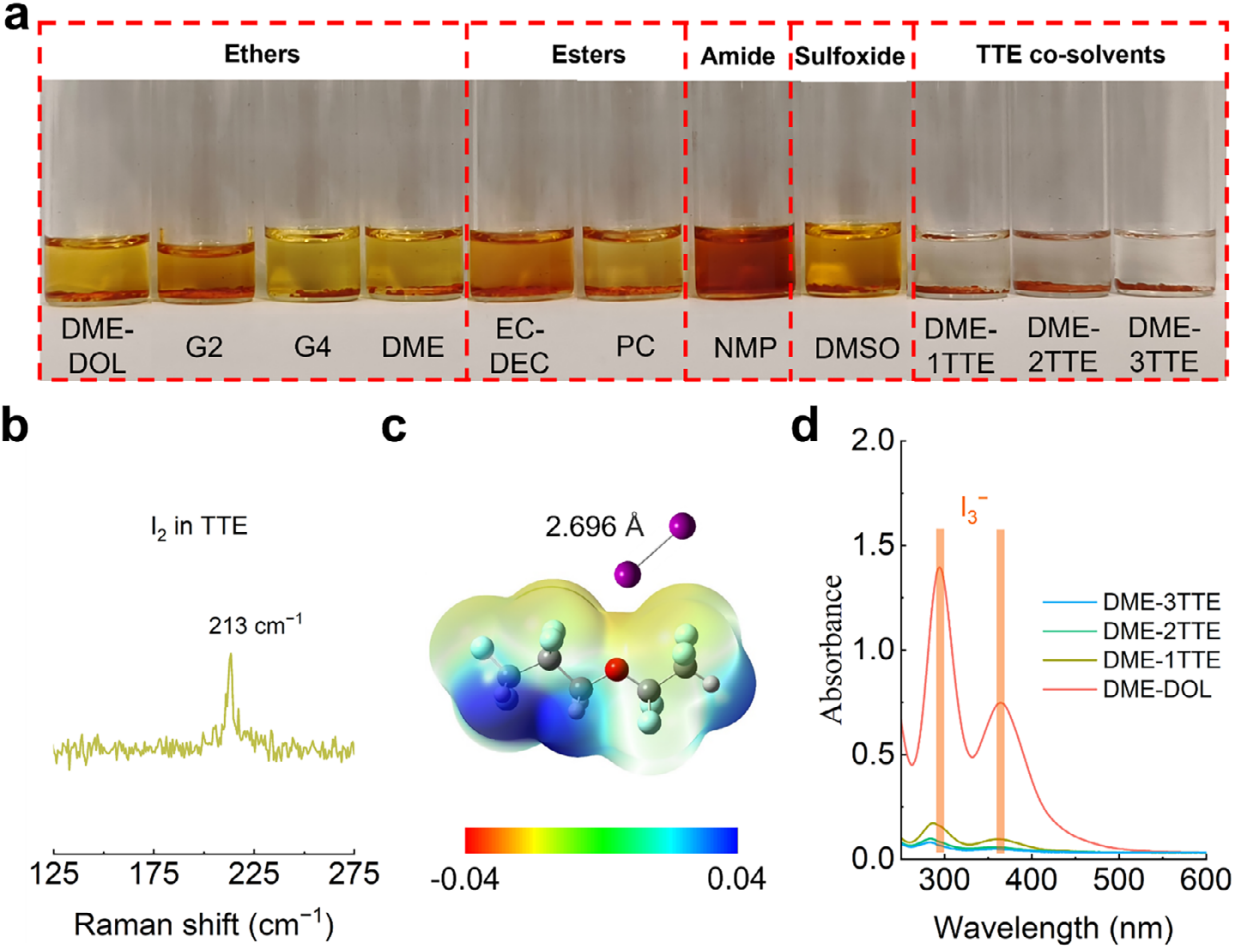
Electrolyte optimization. (a) Digital photo of the dissolution of D·I_2_ in the electrolyte solvents; (b) Raman spectrum and (c) calculated I−I bond length of TTE‐I_2_ complex; (d) UV‐Vis spectra of 10 mg D·I_2_ in 1.5 mL electrolytes after rest for 1 h.

After designing promising host electrodes, we then focus on developing suitable electrolytes to minimize the iodine dissolution. The esters and ethers used in conventional electrolytes typically exhibit strong nucleophilicity and considerable coordination strength toward Lewis acidic species, allowing them to competitively coordinate with ligands in the cathode. This competition is demonstrated in Figure 3a, where D·I_2_ undergoes immediate structural collapse, accompanied by extensive iodine dissolution in most electrolyte solvents, including commonly used ethers like DME, diglyme (G2), tetraglyme (G4), and DME‐DOL, as well as typical carbonates such as ethyl carbonate‐diethyl carbonate (EC‐DEC) and propylene carbonate (PC). Emerging electrolyte solvents, such as amide (N‐methyl‐2‐pyrrolidone, NMP) and sulfoxide (dimethyl sulfoxide, DMSO), also exhibit high dissolution ability for D·I_2_. The high solubility of iodine in these solvents can lead to a severe shuttle effect when used in batteries. Although alkanes (such as CH mentioned above) show weak interaction with iodine, the poor salt‐solvating ability and immiscibility with polar solvents impede their application as electrolyte solvents. By comparison, solvent fluorination can decrease electron density on functional groups due to the electron‐withdrawing effect of fluorine atoms, thereby reducing their affinity for iodine. As a proof of concept, 1,1,2,2‐tetrafluoroethyl‐2,2,3,3‐tetrafluoropropylether (TTE) with a wide electrochemical stable window and Li anode stability was chosen for investigation (Figure S17 and Figure S18). We first explored the interaction between iodine and TTE through a Raman test. The Raman stretching frequency of iodine in TTE appears at 213 cm^−1^, indicating that TTE is a weak ligand in coordinating iodine (Figure 3b). This weak interaction is supported by DFT calculations, which predict an I−I bond length of 2.696 Å in TTE·I_2_ (Figure 3c), only slightly higher than that in CH. Consistently, the UV‐Vis absorption peak at 513 nm, close to the 520 nm observed in CH, further confirms the weak interaction between TTE and iodine (Figure S19).

Since TTE exhibits minimal solubility for lithium salts, it is used as a co‐solvent in combination with DME. We examined how different volume ratios of TTE in the hybrid solvents influence the stabilization of the D·I_2_ electrode. When 50% TTE is mixed with DME (denoted as DME‐1TTE), the structural collapse of D·I_2_ is significantly suppressed, as evidenced by the clear color of the solution after 26‐h immersion (Figure S20). Increasing the TTE content results in even less pronounced color changes. To compare the amount of iodine released into the solvents, UV‐Vis spectroscopy was employed to detect the dissolved iodine species (Figure 3d). In the classic DME‐DOL solvent, strong peaks at 364 nm and 295 nm, attributed to I_3_^−^ ions, are observed. In contrast, these peaks are markedly reduced in DME‐1TTE, and further diminished when the TTE content is increased to 66.7 vol.% (denoted as DME‐2TTE). Increasing the TTE proportion to 75 vol.% (DME‐3TTE) does not result in a significant change, indicating DME‐2TTE is sufficient in immobilizing D·I_2_ electrode. These results demonstrate the feasibility of identifying suitable ligands for electrolyte components based on the I–I bond length determined by Raman spectroscopy.

### 2.3 Electrochemical Performance

The combination of a stable D·I_2_ electrode and a less reactive DME/TTE‐based electrolyte is expected to achieve a synergistic effect in realizing an advanced Li−I_2_ battery. The battery performance was first investigated in electrolytes with various TTE contents. Under DME‐1TTE electrolyte, a fast capacity degradation in the initial cycles is still observed (Figure S21), due to the partial dissolution of D·I_2_ as observed in UV‐Vis results. By comparison, the battery with DME‐2TTE exhibits stable cycling performance. However, a higher TTE ratio leads to the decline of coulombic efficiency (CE), which is ascribed to the limited dissolution of LiNO_3_ additive that is incorporated for boosting I^−^/I° conversion [16, 22]. In addition, the high TTE amount also decreases the ionic conductivity (Figure S22), attributed to incomplete dissociation of the Li salt.

**FIGURE 4:**
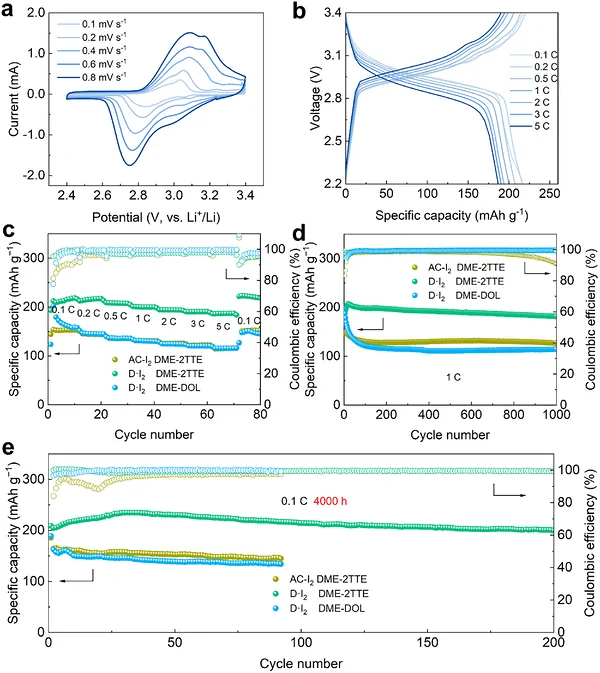
Electrochemical performance. (a) CV curves of the D·I_2_ cathode in DME‐2TTE; (b) voltage profiles of the D·I_2_ cathode in DME‐2TTE at different rates; (c) rate performances of the three battery systems; and their cycling performances (d) at 1 C and (e) at 0.1 C.

With the optimized electrolyte, the electrochemical behavior of D·I_2_ was explored. CV curves in Figure 4a show a pair of distinct redox peaks at around 3.0 V, corresponding to the redox couple of I^−^/I^0^. The small peak at a higher voltage of 3.1 V may be attributed to the conversion of I_3_^−^ back to D·I_2_. Benefiting from strong ligand complexation in the cathode and weak competition from the electrolyte, the battery with the DME‐2TTE electrolyte shows higher capacity at different current densities and satisfactory rate performance ranging from 0.1 to 5 C (Figure 4b,c). A high discharge capacity of 218 and 217 mAh g^−1^ is achieved at 0.1 and 0.2 C, respectively. A capacity of 187 mAh g^−1^ is maintained at 5 C, which corresponds to an 86% capacity retention compared with that at 0.1 C, reflecting the outstanding rate capability enabled by fast kinetics. By contrast, in the classic DME/DOL‐based electrolyte, significantly lower capacities are observed at all tested current densities. To examine the effect of the host electrode, the prepared AC‐I_2_ cathode was used for comparison. Under the same DME‐2TTE electrolyte, the AC‐I_2_ cathode exhibits much lower discharge capacity and worse rate performance than D·I_2_. Furthermore, under optimized DME‐2TTE electrolyte, the D·I_2_ cathode exhibits superior capacity retention of 93.2% after a 24‐h rest, significantly outperforming 61% for the AC‐I_2_ cathode (Figure S23). These results highlight the necessity of concurrently optimizing both the host and the electrolyte to effectively suppress iodine dissolution.

The cycling performance was then investigated at different rates. At 1 C in DME‐2TTE, the D·I_2_ cathode demonstrated a high capacity retention of 87.6% (relative to the peak capacity) after 1000 cycles, retaining a capacity of 182 mAh g^−1^. In contrast, rapid capacity fading is shown during the first 100 cycles in DME‐DOL. The AC‐I_2_ cathode shows a decent capacity retention in DME‐2TTE electrolyte because of weakened electrolyte‐iodine interaction. Nevertheless, a capacity decrease is also observed at initial cycles, and only 131 mAh g^−1^ is retained after 1000 cycles (Figure 4d). The CE of a battery with AC‐I_2_ cathode also exhibits a deterioration in the last several hundred cycles, indicating the continuous loss of active materials due to shuttling. The shuttle effect of iodine species will be more pronounced at low current densities. At 0.1 C, the two battery systems with AC‐I_2_ in DME‐2TTE and D·I_2_ in DME‐DOL show rapid capacity decline (Figure 4e). By contrast, benefiting from the weak competition coordination of TTE toward iodine, the D·I_2_ cathode exhibits stable cycling capacity of above 200 mAh g^−1^ and long cycling stability of over 4000 h. A capacity increase is observed during the initial cycles, which is attributed to an activation process resulting from reduced interfacial resistance and improved electrolyte wettability. As confirmed by in situ electrochemical impedance spectroscopy (EIS) (Figure S24), the gradual decrease in charge transfer resistance enhances active material utilization, leading to a peak capacity of 234.7 mAh g^−1^. This value exceeds the theoretical two‐electron redox capacity of 211 mAh g^−1^ due to the additional pseudocapacitive contribution from the carbon additives (Figure S25). This performance is superior to most reported literature (Table S2). Furthermore, even at an ultra‐high iodine content of 72 wt.%, a decent performance at 0.1 C was also achieved (Figure S26). Similar superiority of DME‐2TTE system was also observed at 0.2 C (Figure S27). To evaluate electrode kinetics, the galvanostatic intermittent titration technique (GITT) was applied. D·I_2_ cathode in both DME‐DOL and DME‐2TTE electrolytes shows faster Li^+^ diffusion behavior than the AC‐I_2_ cathode (Figure S28 ∼ Figure S30), suggesting the advantage of an iodine‐complex cathode over AC‐I_2_ cathode. Furthermore, the D·I_2_ electrode exhibits an average diffusion coefficient of 3.7 × 10^−10^ cm^2^ s^−1^ in DME‐DOL, whereas it increased to 8.7 × 10^−10^ cm^2^ s^−1^ in DME‐2TTE, consistent with its better rate performance in the latter.

### 2.4 Reaction Mechanism

**FIGURE 5:**
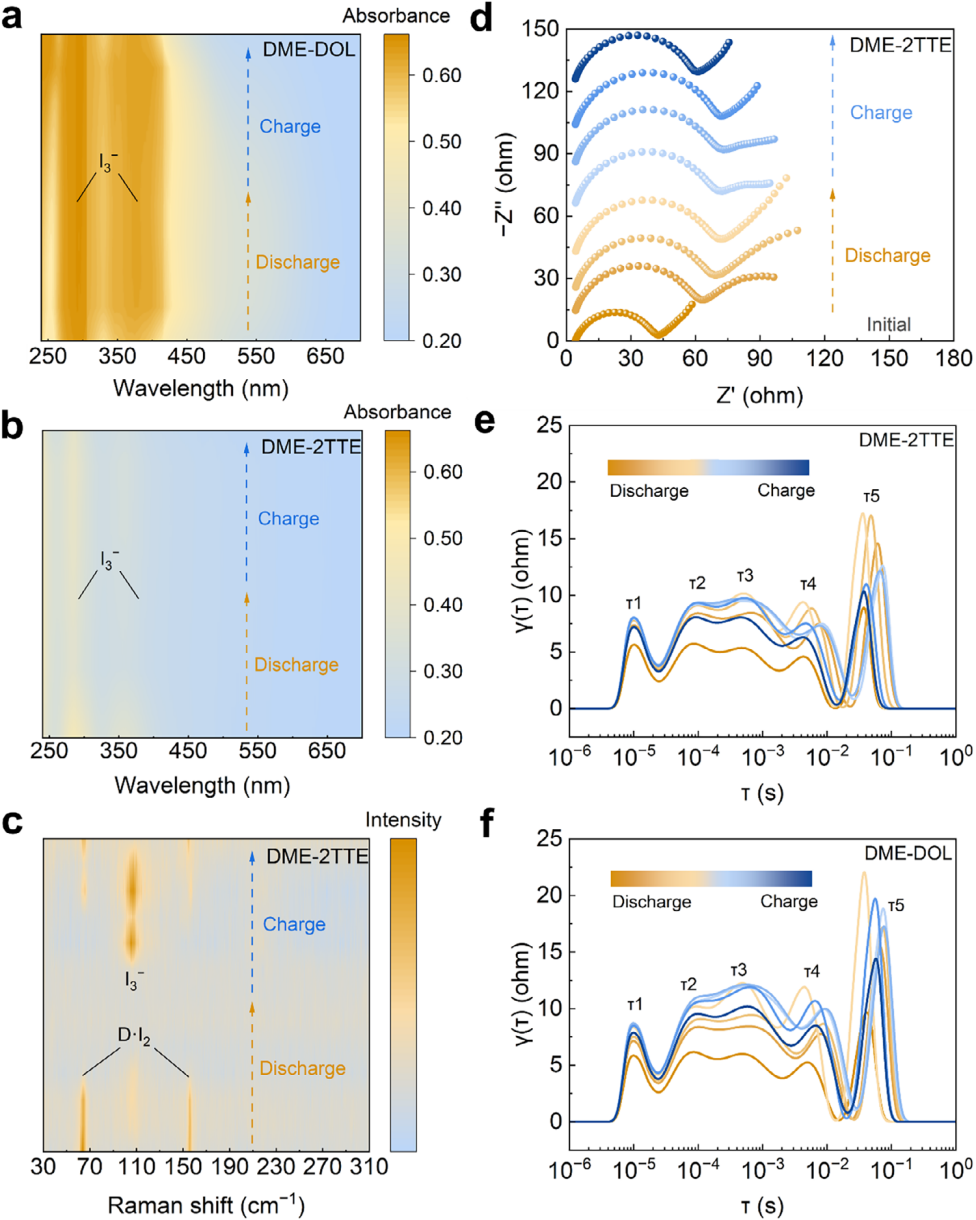
Reaction mechanism. In situ UV‐Vis spectra of D·I_2_ cathode in (a) DME‐DOL electrolyte and (b) DME‐2TTE electrolyte; (c) in situ Raman spectra and (d) in situ EIS spectra of D·I_2_ cathode in DME‐2TTE electrolyte; DRT analysis of D·I_2_ in (e) DME‐2TTE and in (f) DME‐DOL electrolyte.

The reaction mechanism of D·I_2_ in the two electrolytes was investigated through a series of in situ characterizations. In situ UV‐Vis spectroscopy was employed to monitor the generation of polyiodides. Under DME‐DOL electrolyte, the competition from the solvents disrupts D·I_2_ complex and results in substantial formation of I_3_^−^ species, evidenced by the strong absorption peak at 295 and 364 nm during the entire redox process (Figure 5a). By contrast, Figure 5b shows that less I_3_^−^ is generated and diffuses into DME‐2TTE electrolyte, suggesting more stable iodine complexation of D·I_2_, which is attributed to the weak coordination of TTE toward iodine. The structural stability of D·I_2_ in DME‐2TTE is further explored by in situ Raman spectroscopy. As shown in Figure 5c, the peak of I_3_^−^ at around 110 cm^−1^ emerges at the beginning of discharge, which agrees with the UV‐Vis spectra. The formation of a small amount of I_3_^−^ as a liquid‐phase intermediate is beneficial for enhancing reaction kinetics and rate performance. The limited formation and slow release of I_3_^−^ are confirmed by weak UV‐Vis peaks and further supported by its coexistence with D·I_2_ during both discharging and charging in Raman spectra. At the end of the cycle, the typical peaks of D·I_2_ reappear, suggesting the outstanding reversibility of the D·I_2_ cathode under optimal DME‐2TTE electrolyte. In comparison, due to the competition coordination from the electrolyte, the D·I_2_ undergoes rapid dissolution in DME‐DOL based electrolyte, as evidenced by the disappearance of its Raman signal during early discharging (Figure S31). At the end of charging, the absence of a characteristic peak of D·I_2_ indicates this process is irreversible. These observations demonstrate that the competitive coordination disrupts the D·I_2_ structure and leads to capacity degradation.

To acquire a deep understanding of the interface evolution in the battery, in situ EIS combined with distribution of relaxation times (DRT) was employed to analyze the impedance evolution in the time domain. The EIS spectra showed that the interfacial impedance increases during discharge, ascribing to the formation of reduction products (Figure 5d and Figure S32). Upon charging, the impedance exhibits an opposite trend arising from the reversible conversion between D·I_2_ and reduction products. It is noteworthy that the battery with DME‐2TTE demonstrates lower impedance than that with DME‐DOL due to reduced polyiodide formation. The individual impedance contribution was deconvolved through DRT analysis. As shown in Figure 5e,f, the contact impedance corresponding to the peak at 10^−5^ s (*τ*_1_) remains relatively stable during cycling, and no significant difference is observed between the two systems. Clear distinction was observed in the SEI‐related impedance (*τ*_2_) and CEI‐related impedance (*τ*_3_). The cell with DME‐DOL electrolyte exhibits a large increase in impedance, which could be attributed to the generation and migration of polyiodides that corrode electrodes. Charge transfer (*τ*_4_) and mass transport (*τ*_5_) processes also demonstrate large disparity under the two electrolytes. The lower impedance and less growth were observed in DME‐2TTE systems, benefiting the electrochemical kinetics. These results demonstrate that reduced polyiodide leakage leads to lower impedance and ensures efficient charge/mass transfer, thereby improving battery performance.

## 3 Discussion

**FIGURE 6:**
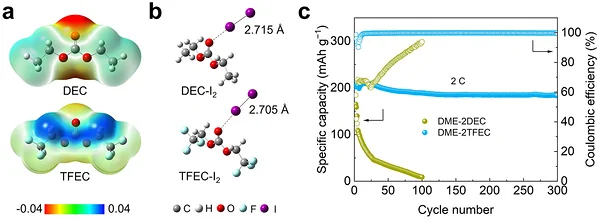
Universal application of the descriptor. (a) ESP mapping of DEC and TFEC; (b) calculated I−I bond length in DEC·I_2_ and TFEC·I_2_; and (c) cycling performance of D·I_2_ cathode in DME‐2DEC and DME‐2TFEC at 2 C after activation at 1 C.

The design principle based on I−I bond length descriptor greatly facilitates stable iodine electrode design. Similar to the D·I_2,_ the longer I−I bond length in the Q·I_2_ indicates the strong anchoring of the host electrode, leading to a stable cyclic performance (Figure S33). Turning to the electrolyte solvent design, a short I−I bond in solvent·I_2_ is preferred for alleviating iodine dissolution. This can be realized through molecular design, such as fluorination, as demonstrated in TTE‐involved electrolytes. The strategy is further extended to carbonate ester solvents. Taking DEC as a representative precursor, bis(2,2,2‐trifluoroethyl) carbonate (TFEC) is obtained through fluorine substitution. The electron‐withdrawing effect of fluorine atoms decreases the nucleophilicity of the carbonyl oxygen, thereby reducing its affinity for iodine (Figure 6a). Figure 6b shows that the I−I bond length decreases from 2.715 Å in DEC‐I_2_ to 2.705 Å in TFEC‐I_2_, indicating weaker coordination of I_2_ to TFEC. Consequently, the cell with the DME‐2TFEC electrolyte exhibits higher capacity and more stable cycling performance than that with DME‐2DEC (Figure 6c), suggesting that the descriptor is universally applicable for optimizing electrodes and electrolytes.

## 4 Conclusion

This study proposes a unified descriptor of I−I bond length that correlates with the coordination strength in iodine‐ligand complexes and presents its application to Li−I_2_ batteries. The bond length increases upon interaction with the ligand, and the degree of elongation depends on the interaction strength, which is also reflected in the linear red shift of the I−I Raman stretching peak. Based on the established principles, ligands with various binding strengths can be employed in different battery components to suppress iodine dissolution and shuttling. Strong ligands with high affinity for iodine should be used as cathode hosts to anchor iodine species, whereas weak ligands are better suited as electrolyte components to mitigate competitive coordination. Accordingly, among the investigated ligands, DABCO was selected to prepare the D·I_2_ cathode, and TTE was employed as a co‐solvent. The battery incorporating the optimal cathode and electrolyte exhibits improved cycling stability with the capacity retention of 87.6% after 1000 cycles. Even at a low current density of 0.1 C, a high discharge capacity and cycling stability of exceeding 4000 h were achieved. The successful extension to carbon ester design further corroborates the design principle. This work provides Li−I_2_ batteries with a comprehensive framework for rational cathode and electrolyte design.

## Author Contributions

**Mengzi Geng**: methodology, data curation, investigation, validation, formal analysis, visualization, writing – original draft, conceptualization. **Yanyan Wang**: investigation, validation. **Fanbin Zeng**: data curation. **Yao Liu**: data curation. **Haijin Ni**: formal analysis. **Bolong Hong**: formal analysis. **Wei Xia**: supervision, resources, writing – review and editing. **Songbai Han**: software, resources, writing – review and editing, funding acquisition, project administration, supervision. **Yusheng Zhao**: resources, writing – review and editing, supervision. **Biao Zhang**: conceptualization, methodology, software, project administration, funding acquisition, resources, writing – review and editing, validation, supervision.

## Conflicts of Interest

The authors declare no conflicts of interest.

## Supporting information



Experimental procedure, characterization and computation methods, electrochemical tests, supplementary note, and supplementary figures/tables are provided in the Supporting Information file [20, 21, 27, 29, 43, 44, 45, 46, 47, 48, 49, 50, 51]. **Supporting File**: anie73430‐sup‐0001‐SuppMat.pdf.

## Data Availability

The data that support the findings of this study are available from the corresponding author upon reasonable request.
