# Supplementary material for: Bond Length as a Unified Descriptor for Stable Iodine Battery
Source: Angew Chem Int Ed Engl. 2026 Jul 2;65(36):e9885697. doi: 10.1002/anie.9885697 (PMC13528457; doi:10.1002/anie.9885697)
Supplement: Supplementary file 1 [file ANIE-65-e9885697-s001.pdf]

Supporting Information

Bond Length as a Unified Descriptor for Stable Iodine Battery

Mengzi Geng[a,b,c], Yanyan Wang[a], Fanbin Zeng[a], Yao Liu[a,b], Haijin Ni[c], Bolong Hong[c], Wei Xia*[b], Songbai Han*[c], Yusheng Zhao*[b], Biao Zhang*[a]

[a] M. Geng, Dr. Y. Wang, F. Zeng, Y. Liu, Prof. B. Zhang
Department of Applied Physics and Research Institute for Advanced Manufacturing
The Hong Kong Polytechnic University
Kowloon, Hong Kong, 999077, China
E-mail: biao.ap.zhang@polyu.edu.hk

[b] M. Geng, Y. Liu, Prof. W. Xia, Prof. Y. Zhao
Eastern Institute for Advanced Study
Eastern Institute of Technology, Ningbo
Ningbo, 315200, China
E-mail: wxia@eitech.edu.cn; yzhao@eitech.edu.cn

[c] M. Geng, H. Ni, B. Hong, Prof. S. Han
Shenzhen Key Laboratory of Solid-State Batteries
Southern University of Science and Technology
Shenzhen, 518055, China
E-mail: hansb@sustech.edu.cn

Experimental Section

Chemicals

Iodine (>99%), hydroiodic acid (HI, 45-50%) 1,4-Diazabicyclo[2.2.2]octane (DABCO, >99%), Cyclohexane (CH, >99.9%), Tetrahydrofuran (THF, HPLC grade), Triethyl phosphate (TEP, >99%), Dimethyl sulfoxide (DMSO, >99%), Pyridine (Py, HPLC grade), Hexamethylphosphoramide (HMPA, >97%), Triethylamine (TEA, AR) and Quinuclidine (Quin, 98%), were purchased from Aladdin. Ethyl carbonate (EC), Diethyl carbonate (DEC), 1,2-Dimethoxyethane (DME), 1,3-Dioxolane (DOL), Diglyme (G2), Tetraglyme (G4), N-Methyl-2-pyrrolidone (NMP), 1,1,2,2-Tetrafluoroethyl-2,2,3,3-tetrafluoropropylether (TTE), Lithium bis(trifluoromethanesulphonyl)imide (LiTFSI) and Propylene carbonate (PC) were purchased from DoDoChem with the purity above 99.8%. Potassium iodide (KI, 99%) and bis(2,2,2-trifluoroethyl) carbonate (TFEC, >98%) were from Macklin. Active carbon (AC, YP80F), Ketjen Black ECP-600JD (KB) and Polytetrafluoroethylene powder (PTFE) were provided by Canrd.

Preparation of active materials and cathodes

$Q{\cdot}I_2$ and $D{\cdot}I_2$ were prepared by dispersing and mixing iodine and Quinuclidine or DABCO in ethanol solution with excess iodine. Then the solid product was filtered and washed with DI water several times to remove unreacted materials, and vacuum dried at 60 °C overnight. AC-I_2 was prepared by milling iodine and AC with a mass ratio of 1:1, and then sealed and heated at 130 °C for 4 hours.

$D{\cdot}I_2$ cathode was prepared by mixing 50 wt.% active material, 30 wt.% AC, 10 wt.% KB in isopropanol with 10 wt.% PTFE as binder. The mixture was stirred until homogeneous and then loaded on the carbon cloth with a diameter of 12 mm. AC-I_2 cathode was prepared by mixing AC-I_2, KB and PTFE with a mass ratio of 7:2:1 in isopropanol. The iodine loading in all samples was controlled to about 1 mg cm^{-2} for fair comparison, and the capacity and current density were calculated based on iodine.

Preparation of electrolytes

1 mol L^{-1} (1 M) LiTFSI in DME-DOL (v/v, 1:1) with 0.1 M $LiNO_3$ was used as conventional ether electrolyte. DME/TTE-based electrolytes were prepared by dissolving 1 M LiTFSI in DME/TTE mixtures with volume ratio of 1:1, 1:2 and 1:3 (DME-xTTE, x = 1, 2, 3) and 0.1 M $LiNO_3$ was added as an additive. In DME-3TTE electrolyte, the concentration of $LiNO_3$ was 0.05 M due to its low solubility. Ester electrolytes of 1 M LiTFSI in DME-2TFEC or in DME-2DEC with 0.1 M $LiNO_3$ were also prepared similarly.

Materials characterization

Scanning electron microscopy coupled with an energy-dispersive X-ray spectrometer was performed on a field-emission SEM (FESEM, JEOL-JSM7610) for morphology and element distribution detections. X-ray diffraction (XRD) and pair distribution function (PDF) data were collected on Malvern Panalytical Empyrean with Cu and Ag as the source of radiation,

respectively. Iodine weight fraction in active materials was determined by Inductively coupled plasma-Mass Spectrometry (ICP-MS) using Agilent 7800. Attenuated Total Reflectance Fourier Transform Infrared Spectroscopy (ATR-FTIR) test was conducted on Nicolet iS5. Thermogravimetric (TG) curves were collected under a nitrogen atmosphere from room temperature to 600 °C with a heating rate of 10 °C min^{-1}.

UV-Vis spectra

UV-Vis spectra were recorded using a Shimadzu UV-2550 spectrophotometer. A quartz cell was applied to conduct in situ experiments. The discharging-charging process was controlled by the LAND battery testing system. The UV-Vis data were collected every 4~6 min.

Raman spectra

Raman tests were performed on WITec alpha300R with an excitation wavelength of 532 nm. The stretching frequency of I−I in the series of solvent ligands was detected by simply dissolving iodine into the solvents except HMPA and TEA. The Raman spectra of HMPA·I_2 and TEA·I_2 were obtained by distributing them in CH. In situ Raman experiments were conducted on the coin cell with a quartz window.

Electrochemical impedance spectra

Electrochemical impedance spectroscopy (EIS) was determined on BioLogic 200 electrochemical workstation. A frequency range of 10^5 to 100 mHz and an alternating current voltage amplitude of 10 mV were applied. Ionic conductivities of the prepared electrolytes were measured in symmetric batteries by using stainless-steel electrodes with the diameter of 15.8 mm, and polyethylene (PE) separator. In situ EIS experiments were conducted at 0.15 C with EIS data collected every 2 h, and a 10-min rest was applied before each measurement. Distribution of relaxation times (DRT) analyses were performed by using DRTtools [1,2].

Electrochemical measurements

Battery assembly was operated in glovebox with both oxygen and water content below 0.1 ppm. Iodine complex worked as cathode, and a lithium foil and PE membrane served as anode and separator, respectively. The batteries were tested on NEWARE and Land battery testing systems at room temperature. A cut-off voltage of 3.4 V was applied for cells with DME/TTE electrolytes while 3.25 V for cells with other electrolytes to avoid side reactions at high voltages. Cyclic voltammetry (CV) and linear scanning voltammetry (LSV) were conducted on BioLogic 200 electrochemical workstation. The galvanostatic intermittent titration technique (GITT) tests were performed at 0.1 C with a 20-min charging/discharging process followed by a 4-hour relaxation. The Li^+ diffusion coefficient was calculated based on

$$D = \frac{4}{\pi\tau}\left(\frac{m_B V_m}{M_B S}\right)^2 \left(\frac{\Delta E_s}{\Delta E_t}\right)^2 \quad \text{(eq S1)}$$

where τ is the duration of the current pulse, m_B is the mass of active material, V_m and M_B are the molar volume and molar mass, respectively, S is the contact area, ΔE_s is the steady-state voltage change, and ΔE_t is the voltage change during the constant current pulse.

Theoretical calculation

All DFT calculations were performed in Gaussian software package [3]. Typical B3LYP functional with def2TZVP basis set was employed to describe the electron interactions [4]. Grimme D3(BJ) was applied for weak van der Waals dispersion correlation [5]. The binding energy between molecule A and B was calculated based on $E=E_{A+B} – E_A – E_B$, where E_{A+B} is their total energy and E_A and E_B are the energy of molecule A and B, respectively.

Supplementary Notes

Supplementary note 1

The HOMO and LUMO, as frontier molecular orbitals, provide a useful approximation for describing chemical reactions. The interaction strength between orbitals is influenced by their shape (s, p, d, etc.), principal quantum number (e.g., 2s, 3s, 4s), symmetry and directionality (σ, π, steric constraints), and relative energy levels. As the HOMO of the ligand approaches the LUMO of iodine in energy, as shown in **Figure S3**, the strength of the dative covalent interaction between them increases due to improved energy matching. Similar to the formation of a covalent bond, this interaction leads to a lowering of the HOMO energy and a raising of the LUMO energy, with the magnitude of these shifts positively correlated with the interaction strength. Consequently, the electron transition from the π* orbital to the σ* orbital requires relatively higher energy.

Supplementary note 2

The compositional and structural differences between the dative covalent complex DABCO·I_2 (D·I_2) and the reported ionic salt DABCO·HI (D·HI) were investigated. The bonding characteristics of precursors of DABCO with I_2 or HI were examined by Raman and FTIR spectroscopy and shown in **Figure S15** and **Figure S16**. In the dative covalent complex D·I_2, the N···I interaction is clearly evident as a band at 64 cm^{-1}. By contrast, pronounced N−H vibrational modes are observed in D·HI, indicating the formation of a tertiary ammonium ion. Moreover, due to their distinct bonding types, the preparation method of D·HI also differs from D·I_2. The D·I_2 can also be synthesized in aqueous solution whereas D·HI needs to be prepared in solvents with low solvation ability. As a typical iodide salt, D·HI is air-sensitive and will hydrolyze in water. The hydrolysis products were verified by UV-Vis spectra in **Figure S16b** where the UV-Vis peak at 225 nm attributed to I^- was observed. This is different from the I−I vibration in D·I_2, highlighting the disparate chemical states of iodine in these two materials.

Supplementary Figures

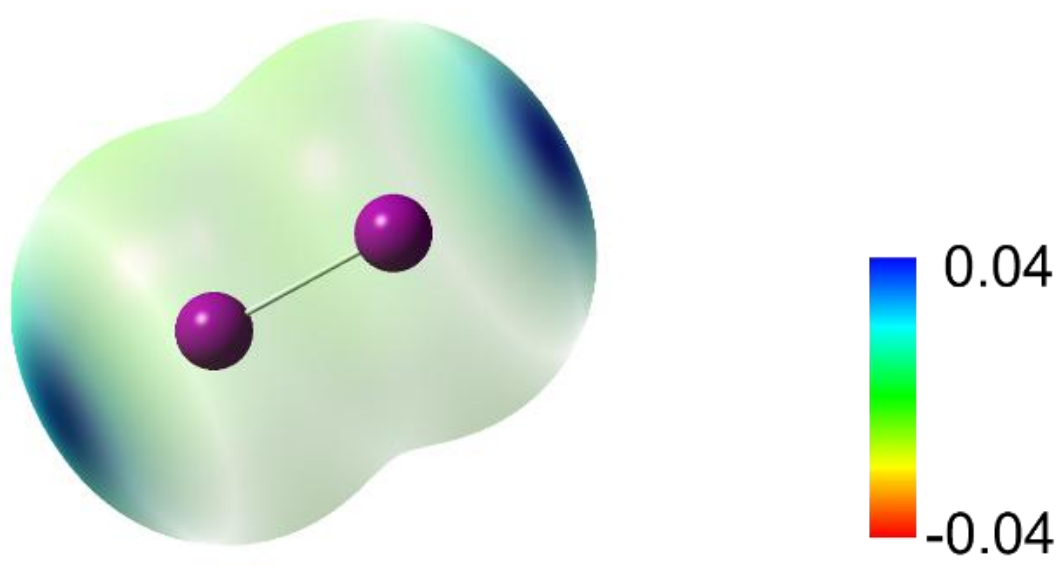

Figure S1. ESP mapping of I_2 molecule.

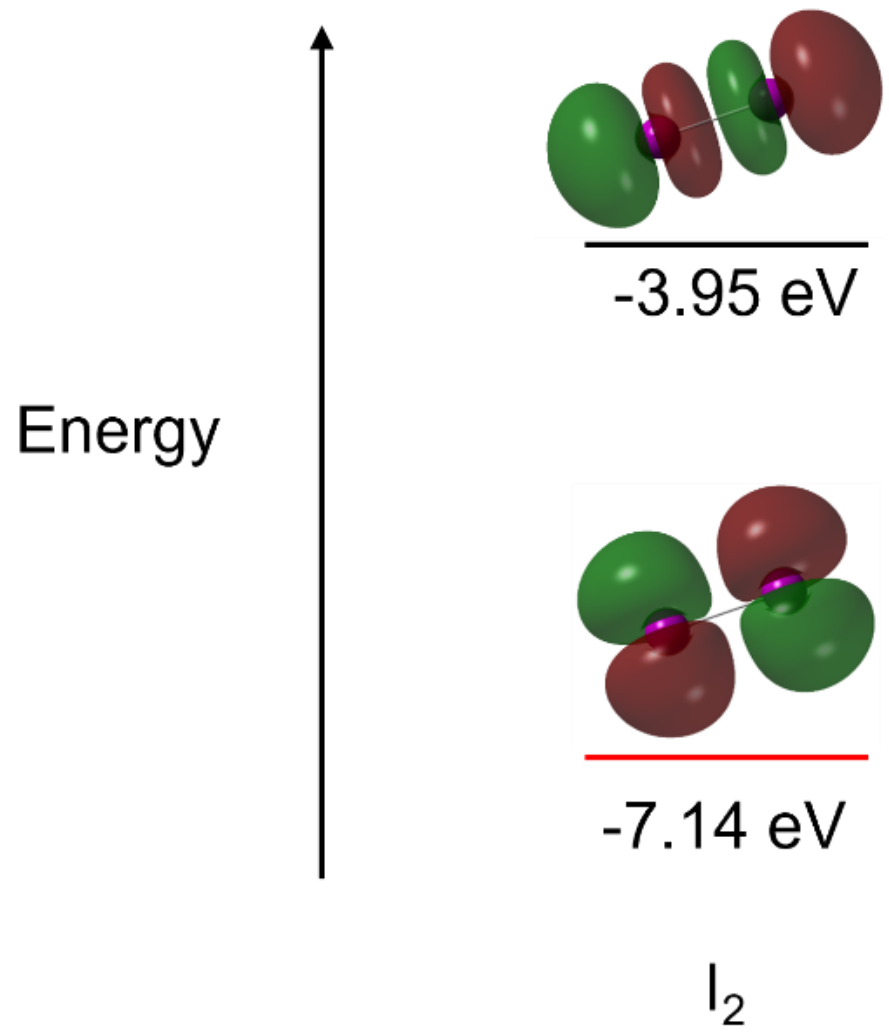

Figure S2. The distribution of the LUMO and the HOMO of I_2 molecule.

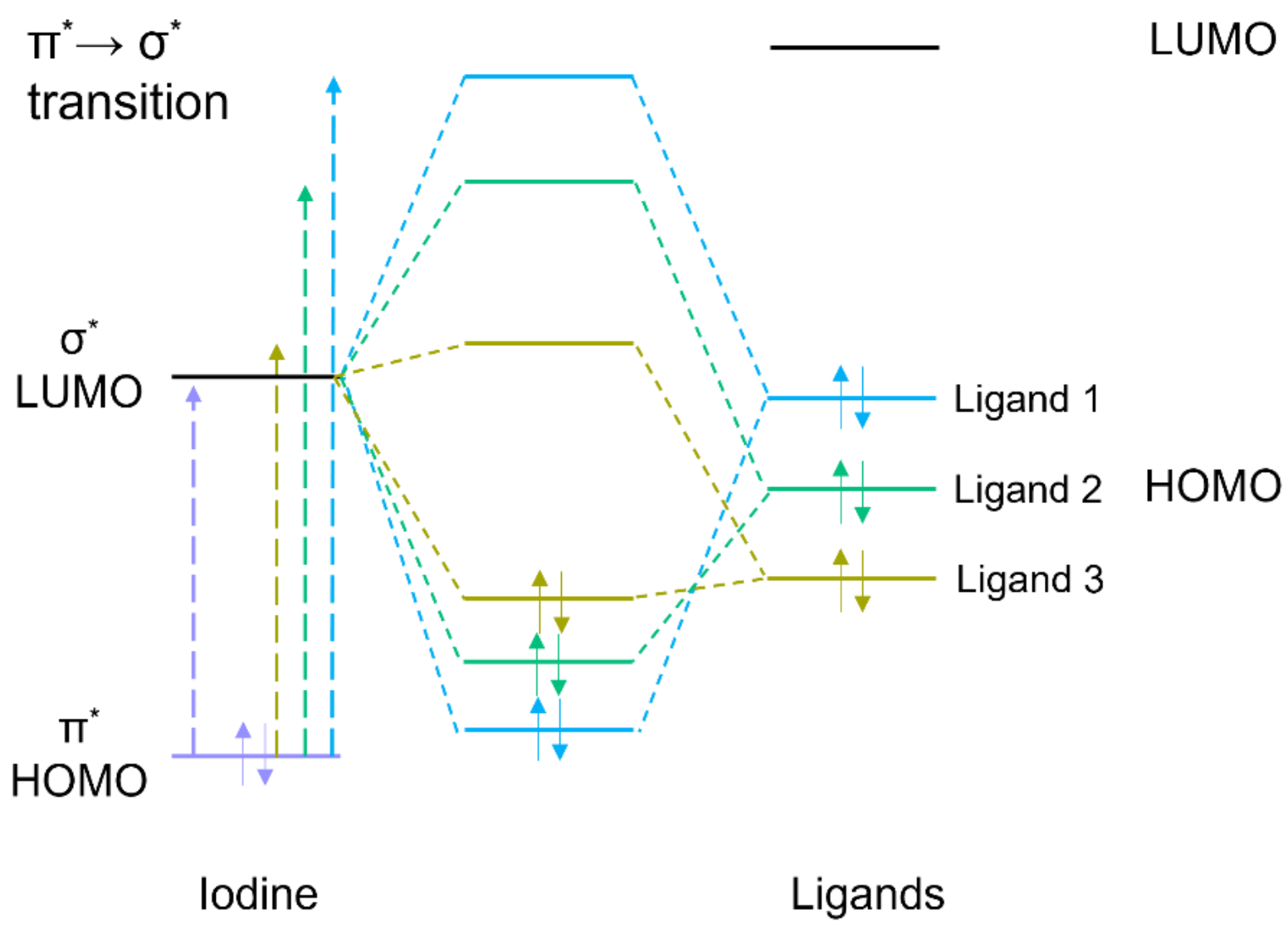

Figure S3. Illustration of the orbital interactions and electron transition processes between iodine and ligands.

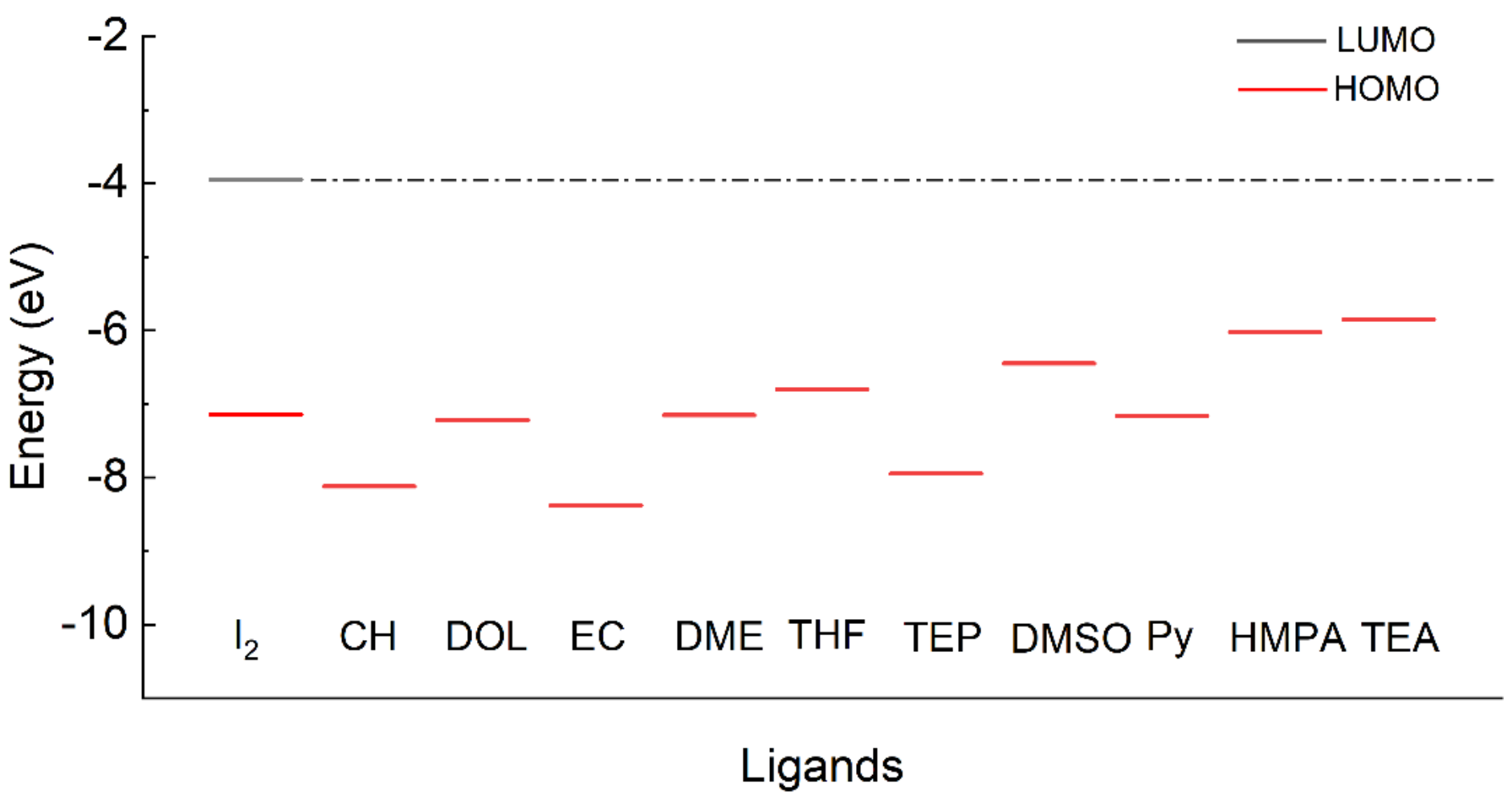

Figure S4. The energy level of I_2 molecule and ligands.

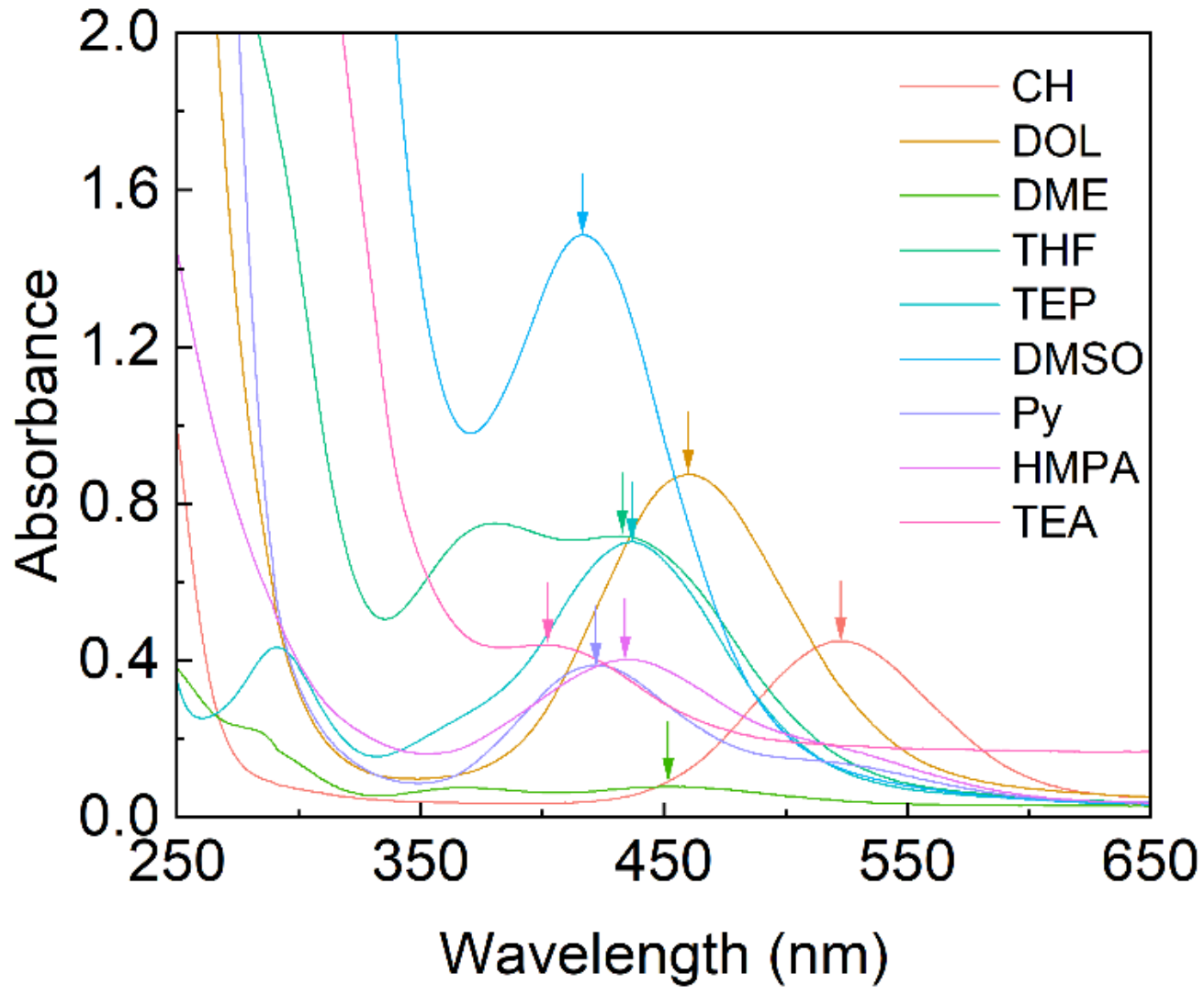

Figure S5. UV-Vis spectra of iodine in different ligands (~1 mM mL^{-1}).

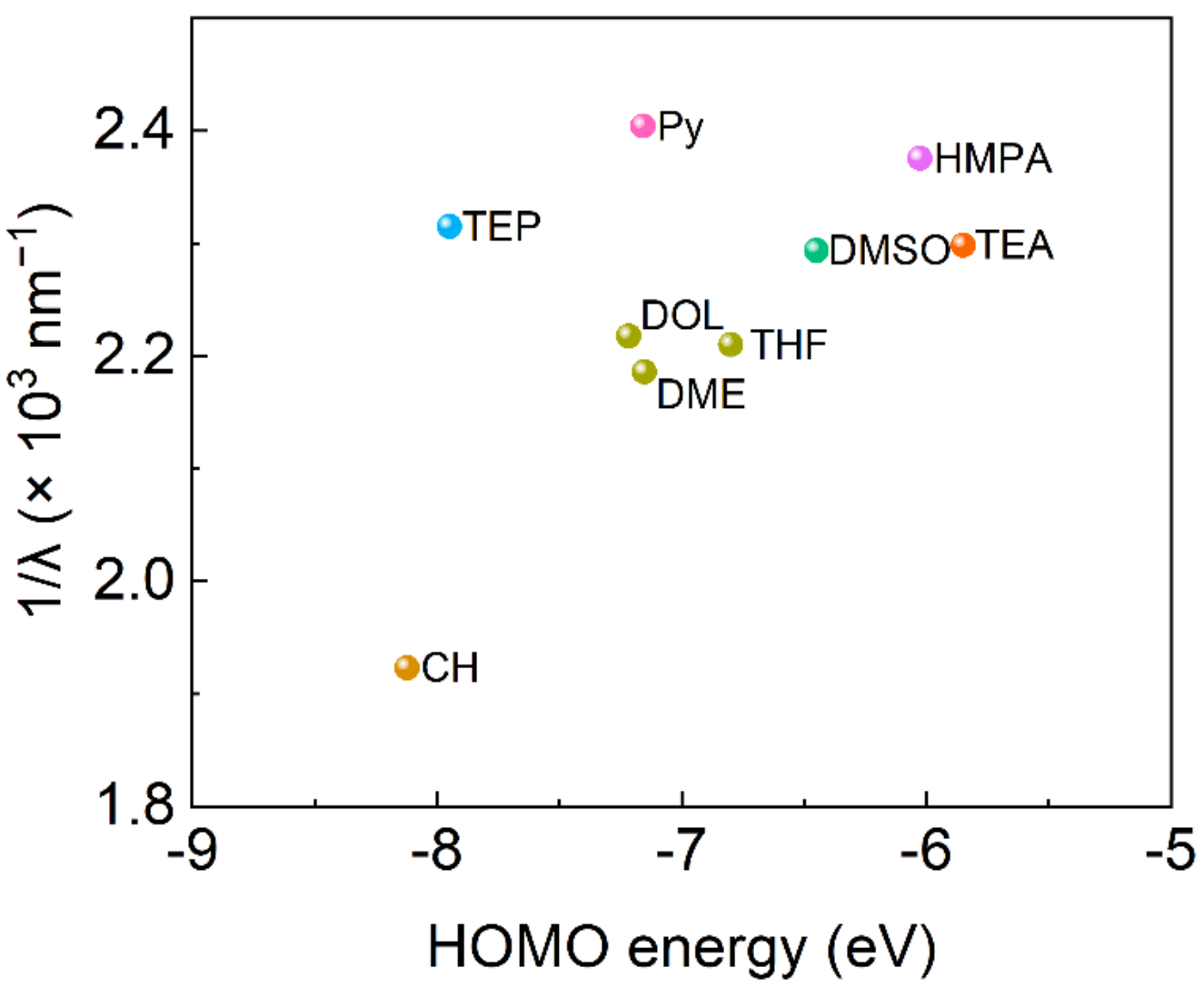

Figure S6. The correlation between the excitation energy of iodine and the HOMO energy level of ligands.

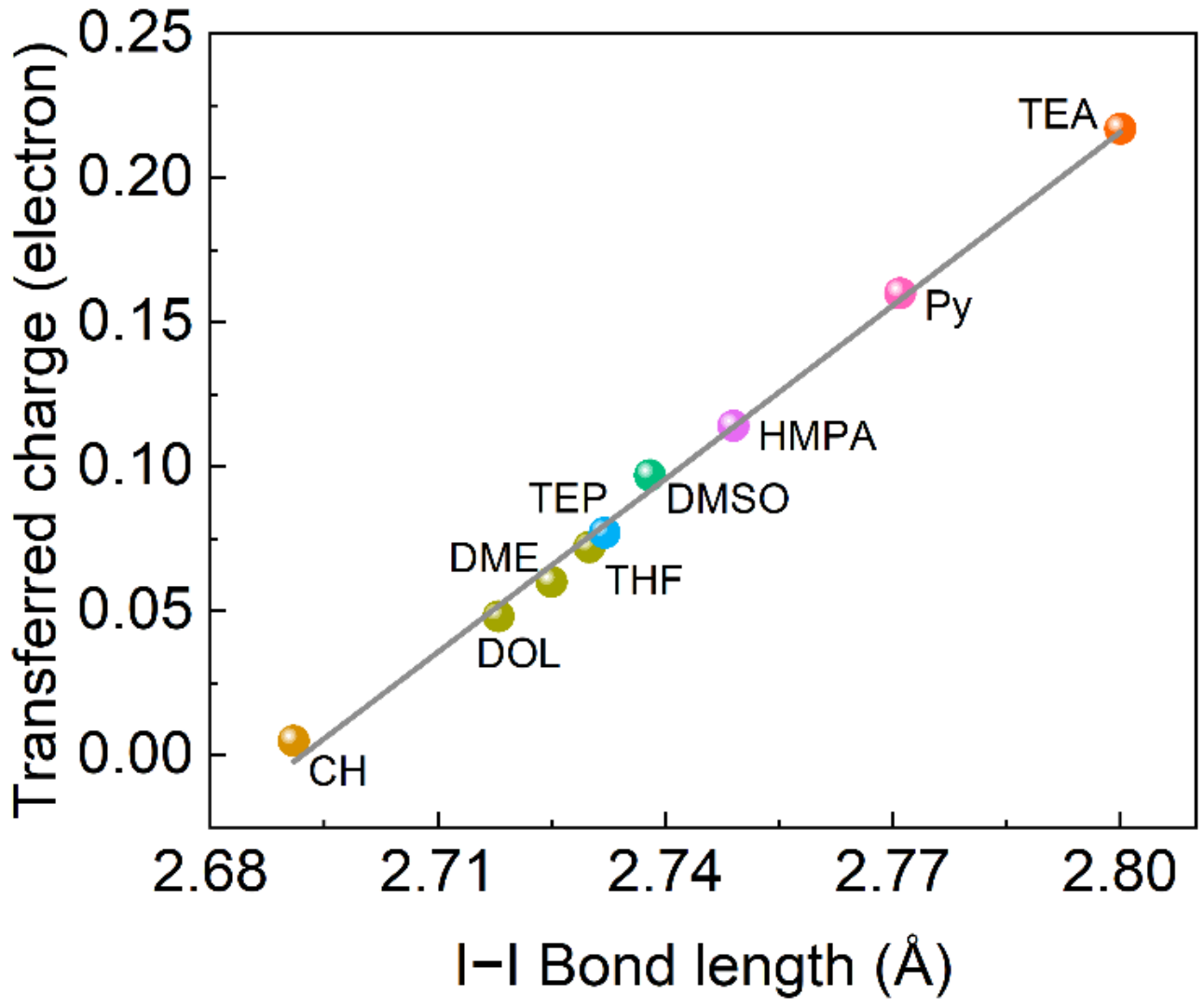

Figure S7. The relationship of transferred charge with I−I bond length in iodine complexes.

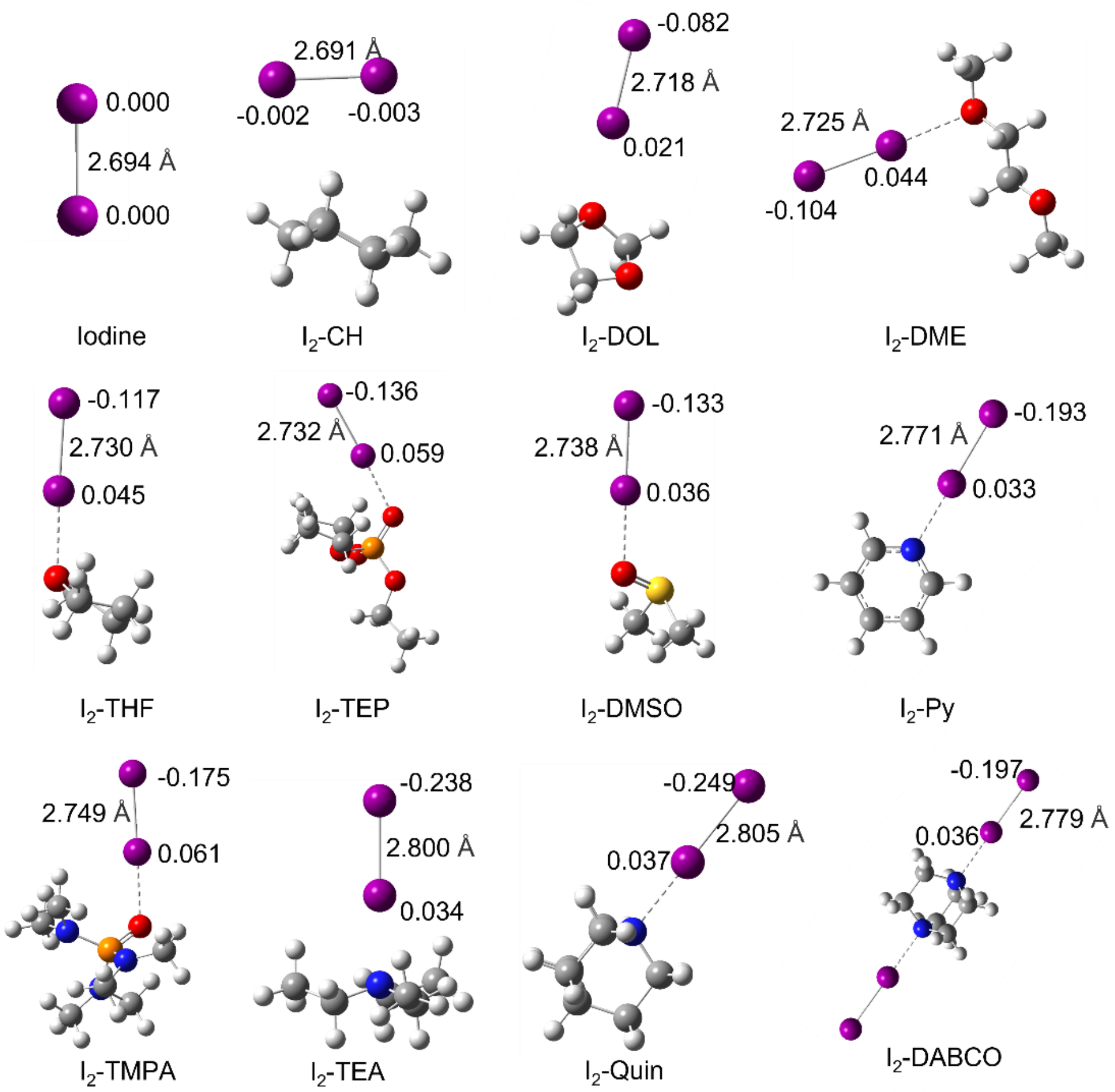

Figure S8. I−I bond length and Mulliken charge in iodine complexes.

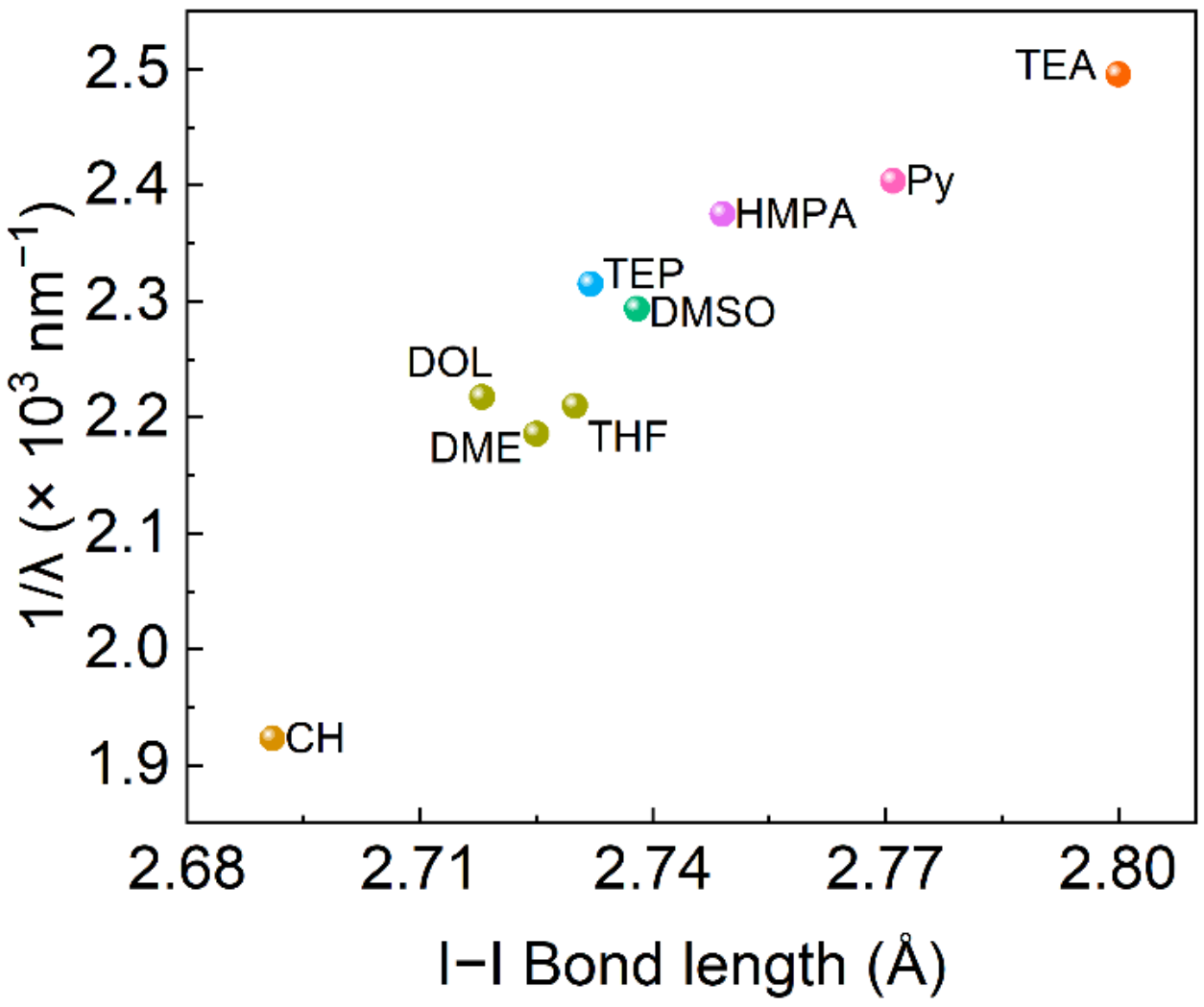

Figure S9. The correlation between the excitation energy and I−I bond length in iodine complexes.

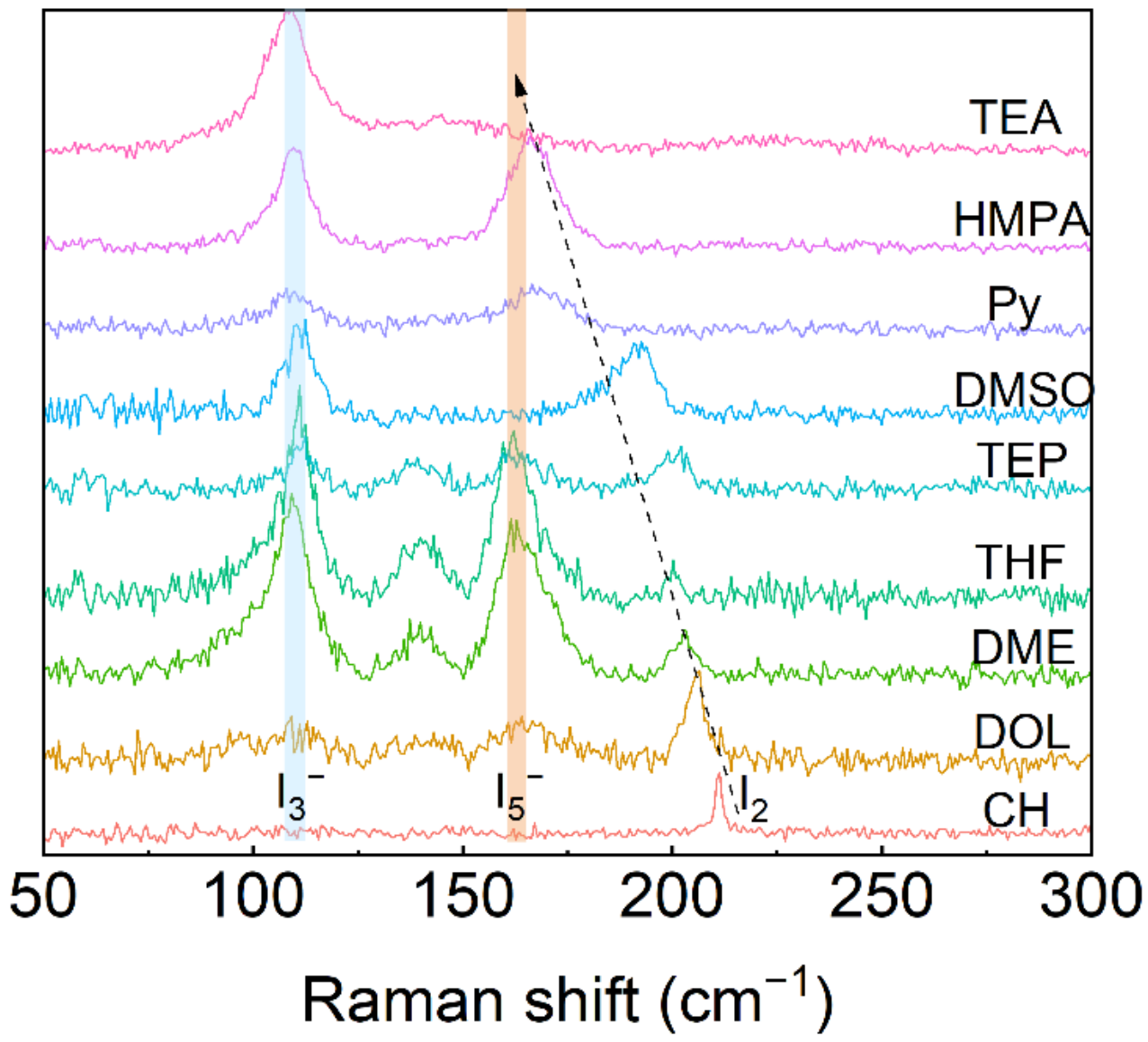

Figure S10. Raman spectra of iodine in different ligand solvents.

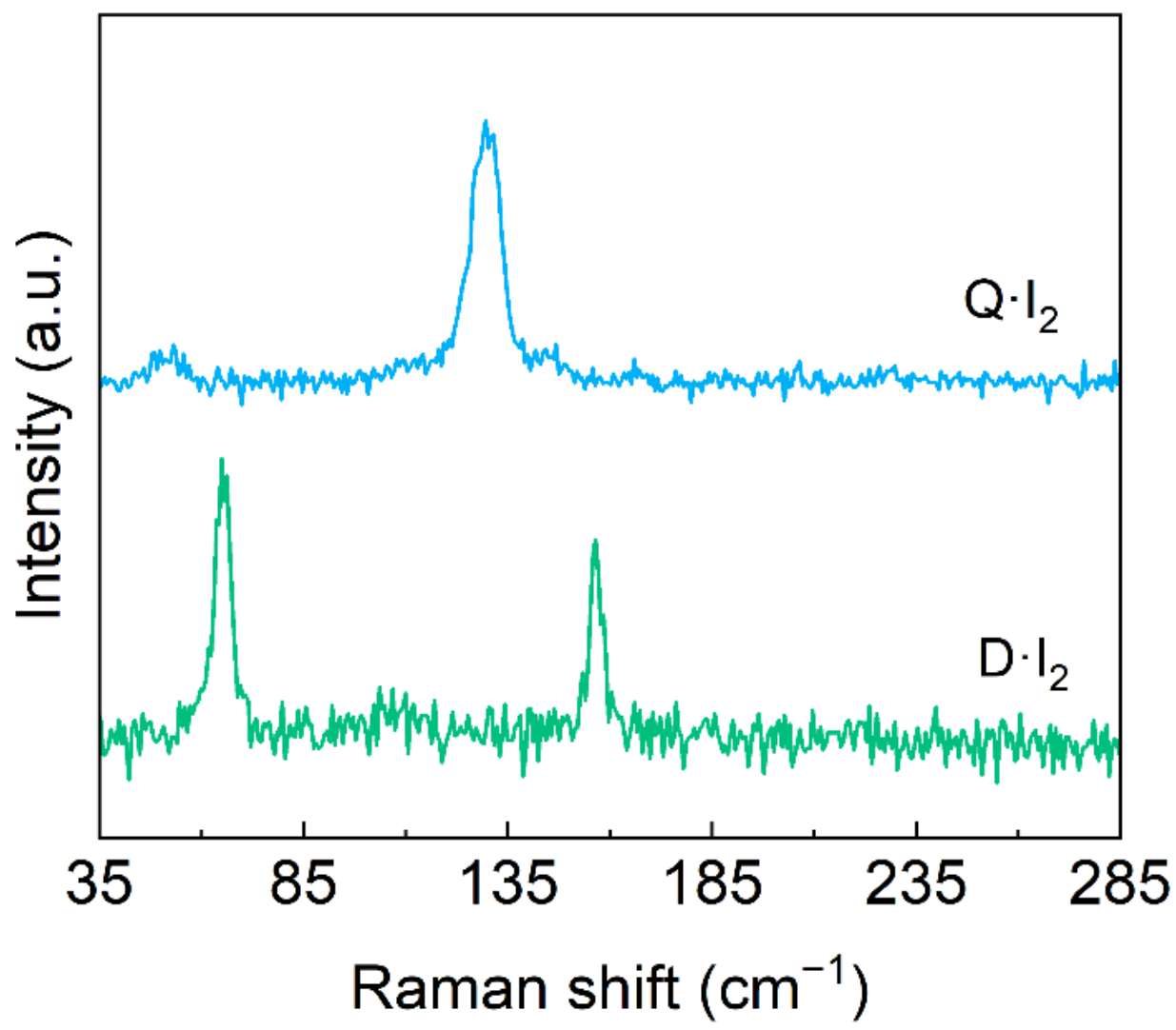

Figure S11. Raman spectra of D·I_2 and Q·I_2 at low vibrational frequency.

Mass of D·I_2 (mg)	Constant volume (mL)	Element concentration in test solution (ug/L)	Dilution ratio	Element content (wt.)
0.0517	25	168.48	10000	81.47%

Mass of Q·I_2 (mg)	Constant volume (mL)	Element concentration in test solution (ug/L)	Dilution ratio	Element content (wt.)
0.0490	25	100.21	10000	51.63%

Figure S12. ICP-MS results of D·I_2 and Q·I_2.

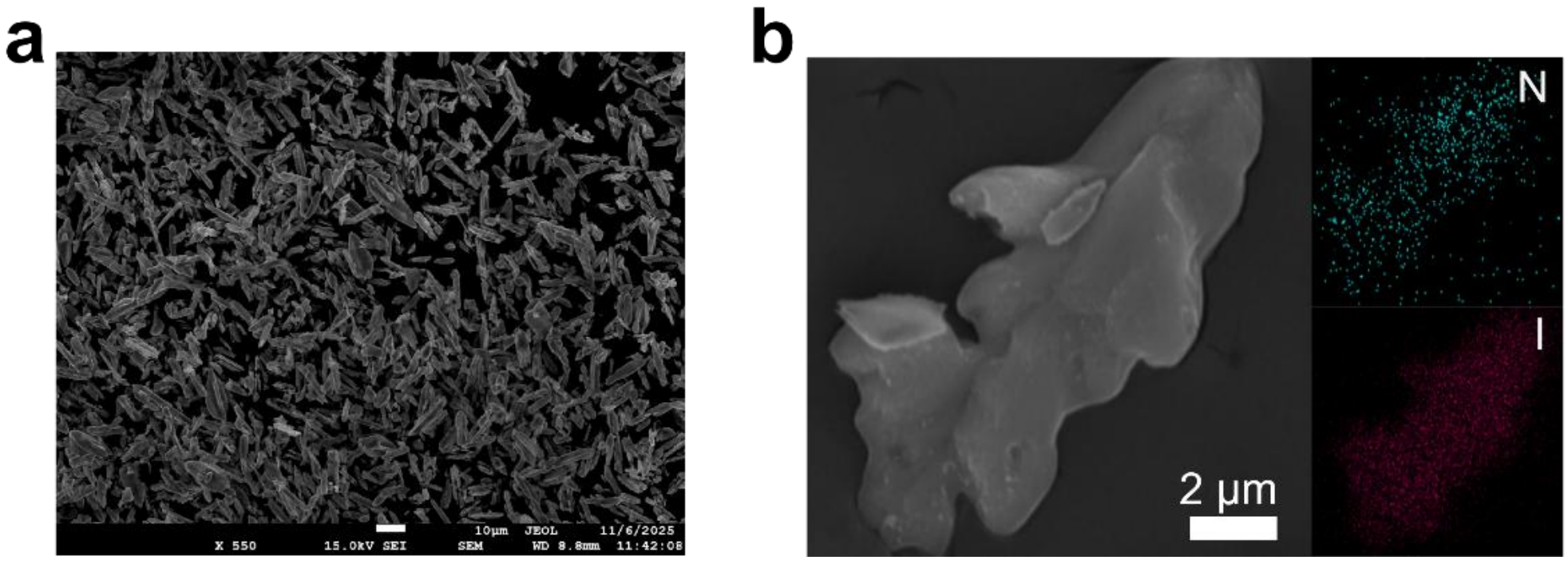

Figure S13. (a) SEM and (b) EDS images of $D{\cdot}I_2$.

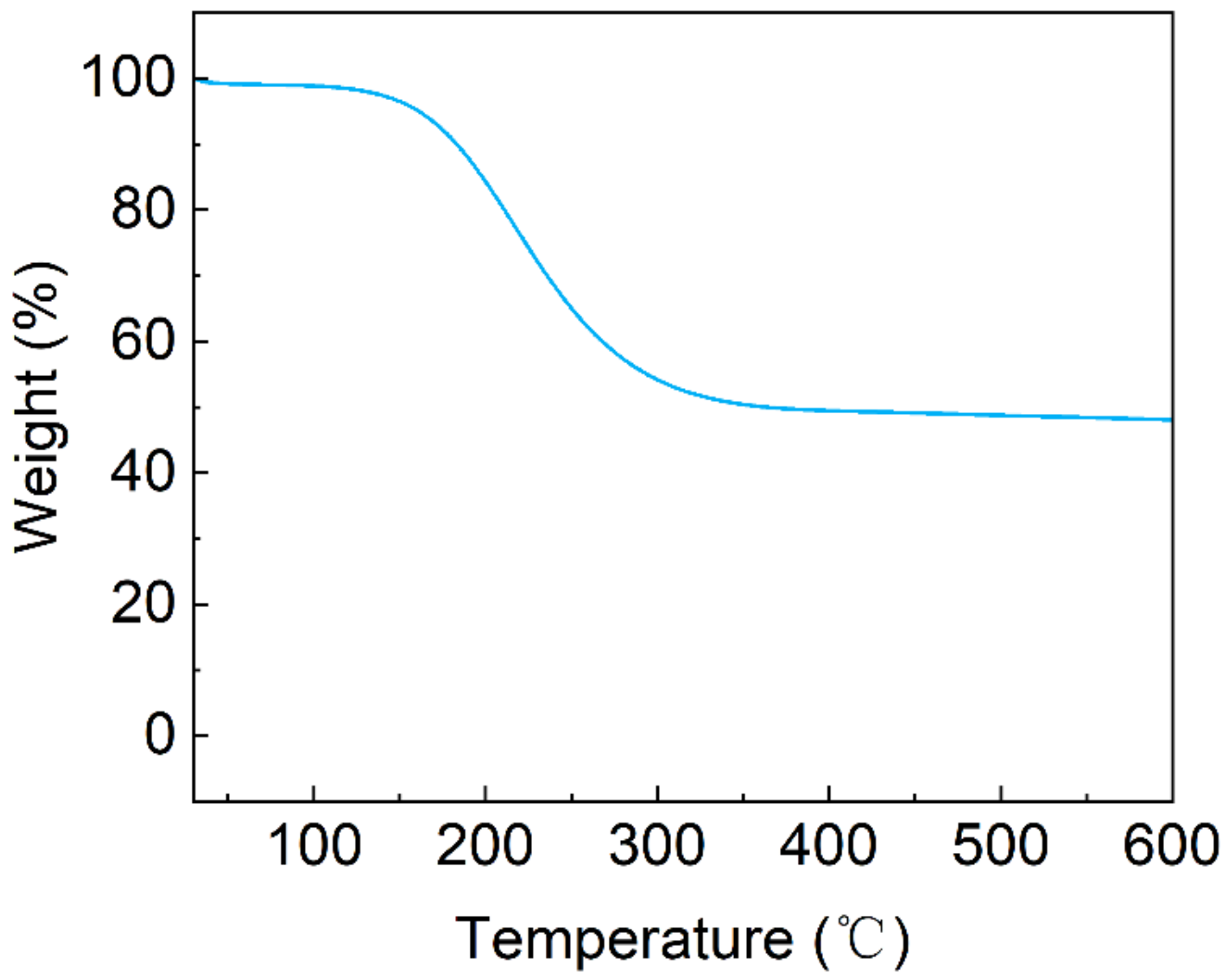

Figure S14. TG curve of AC-I_2.

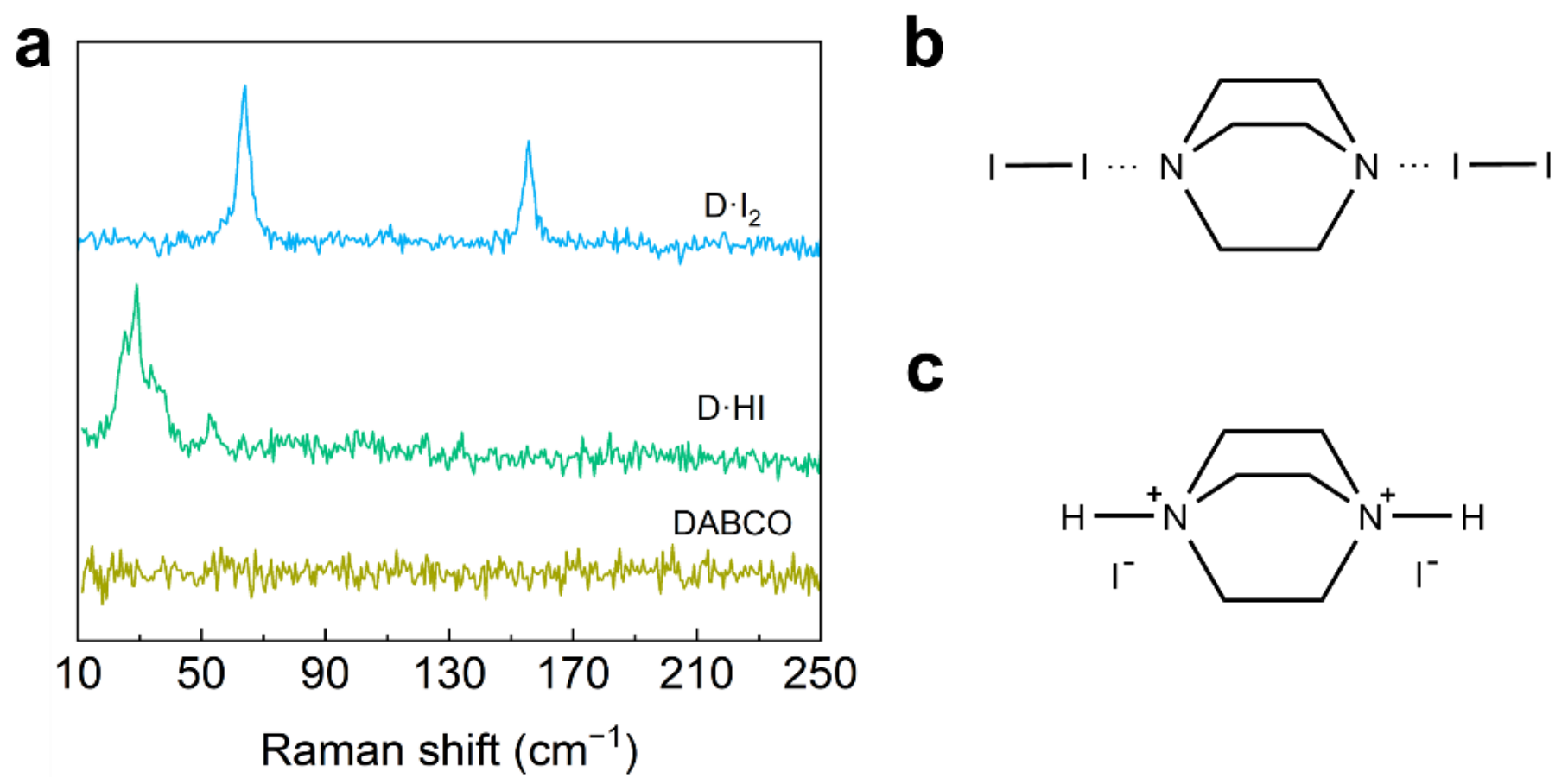

Figure S15. (a) Raman spectra of DABCO, D·HI and D·I_2; illustration of the structure of (b) D·I_2 and (c) D·HI.

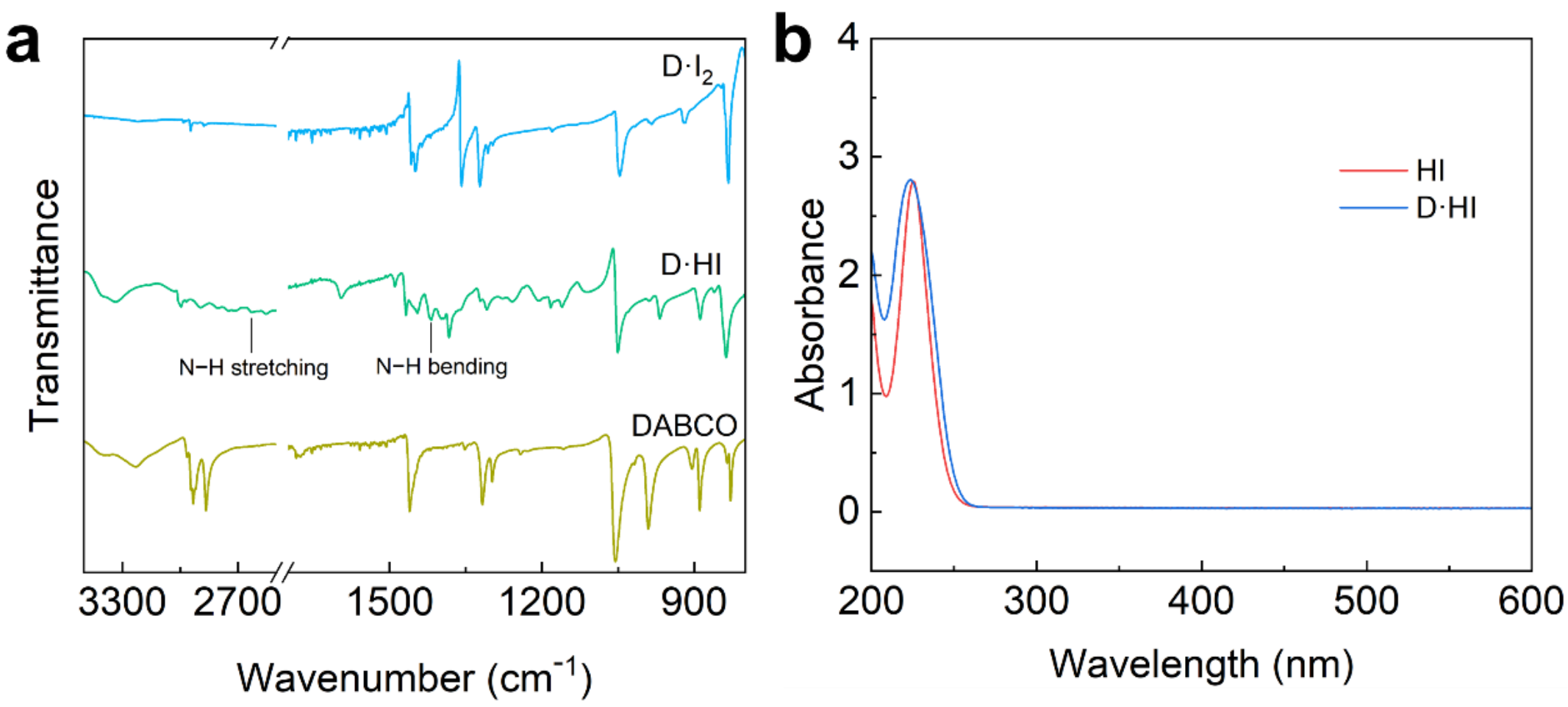

Figure S16. (a) ATR-FTIR spectra of DABCO, D·HI and D·I_2 and (b) the UV-Vis spectra of D·HI and HI in water.

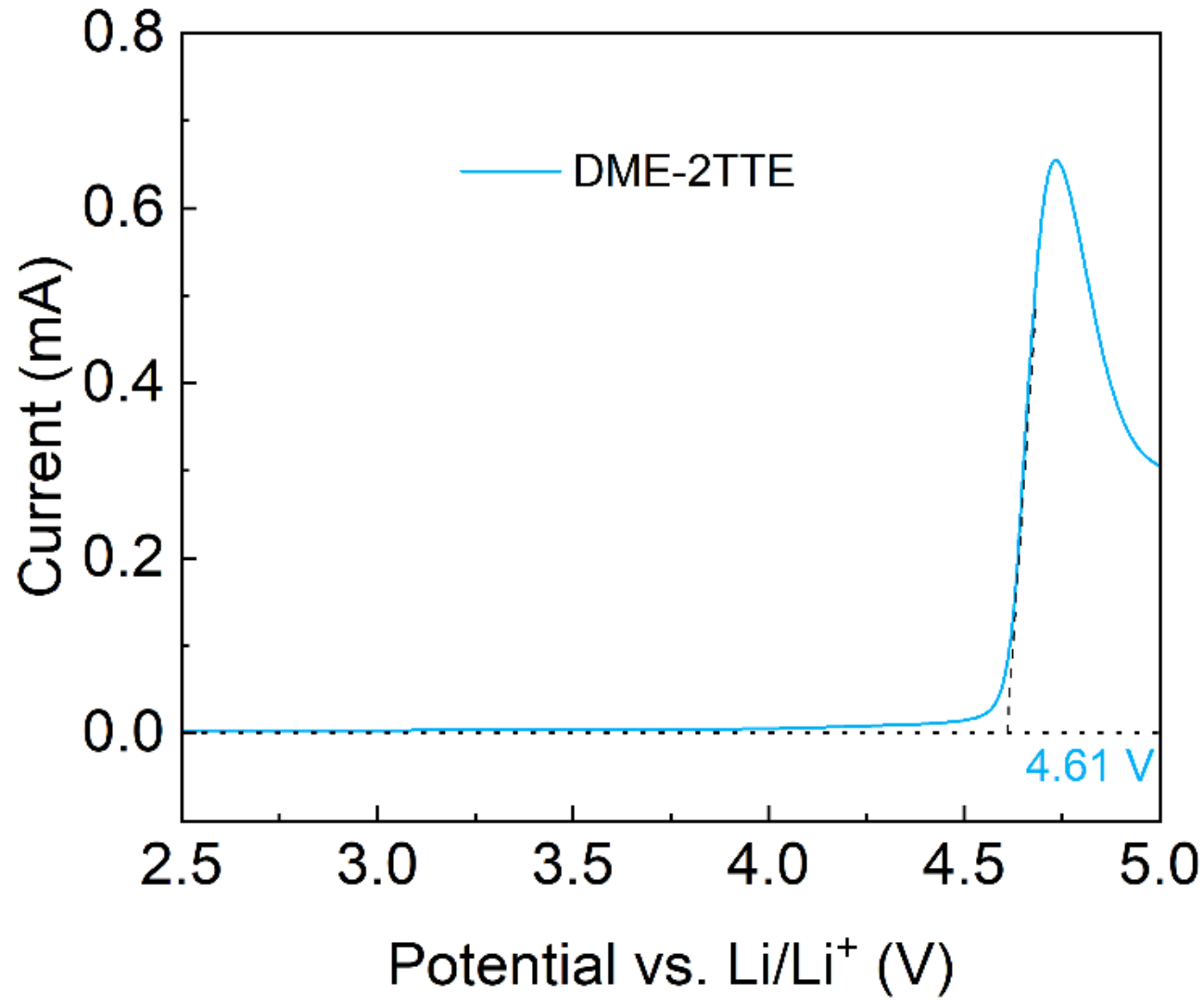

Figure S17. Oxidation stability of the electrolyte with DME-2TTE co-solvent.

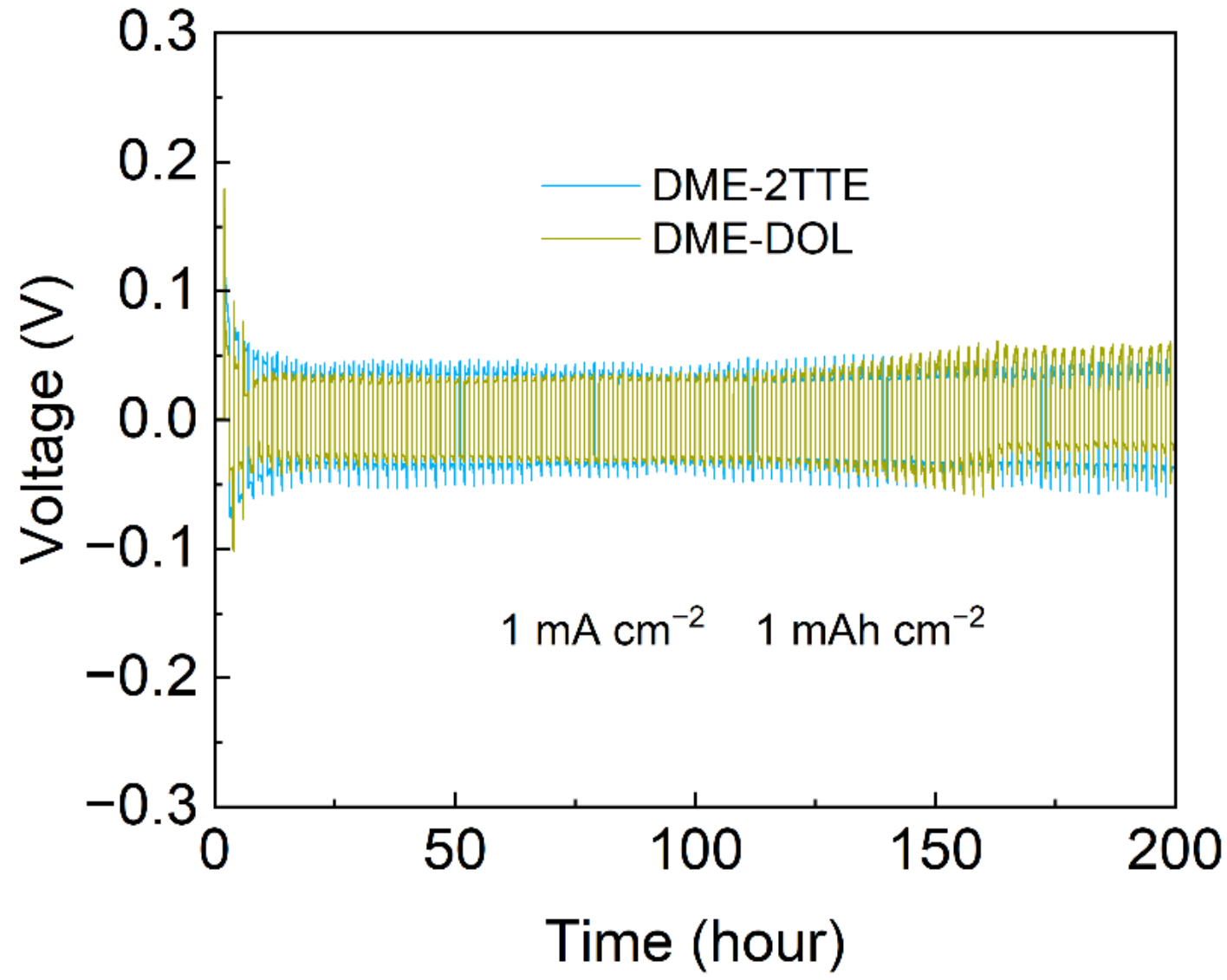

Figure S18. Li anode stability of the electrolytes with DME-2TTE and DME-DOL solvent.

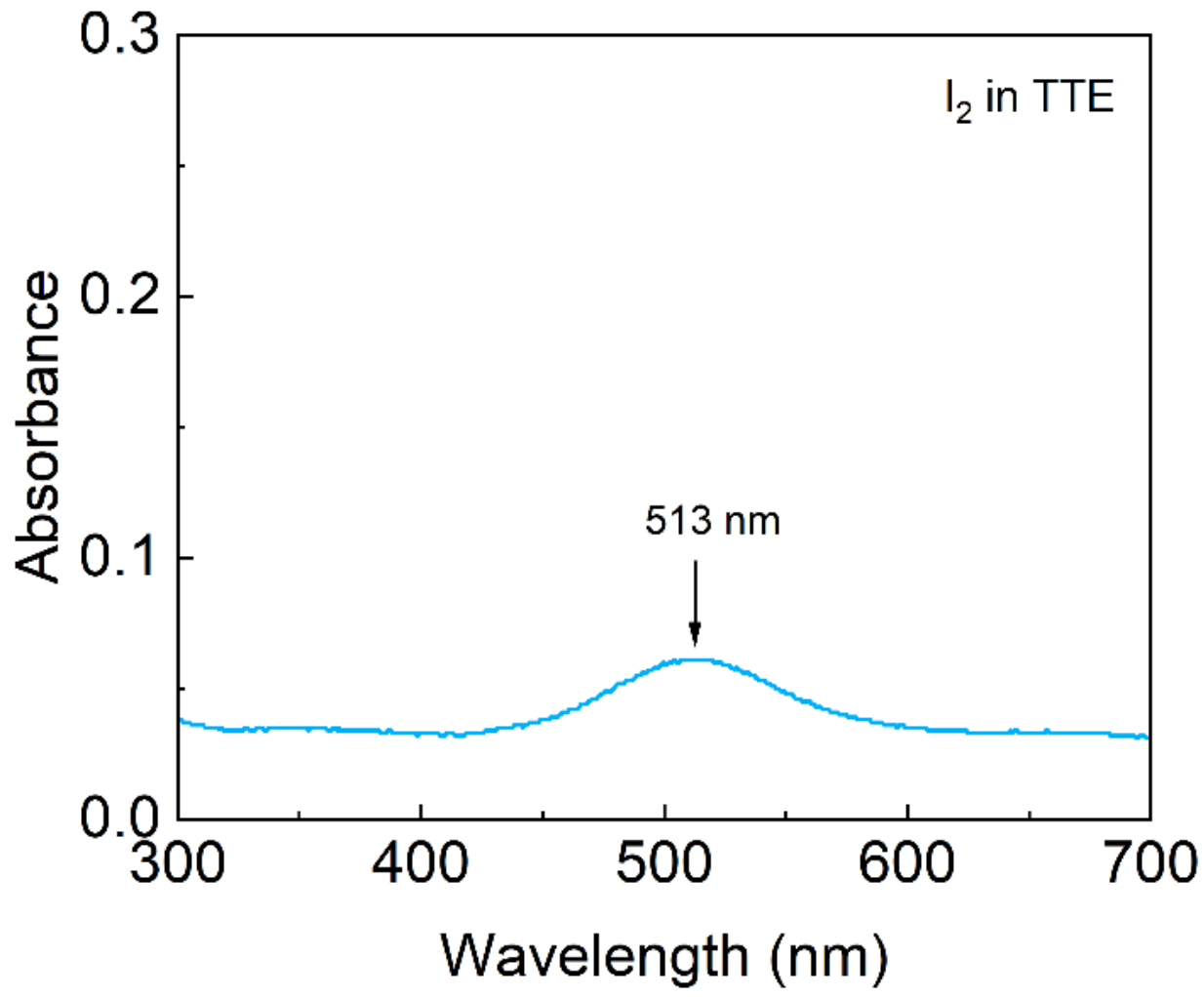

Figure S19. UV-Vis spectrum of iodine in TTE.

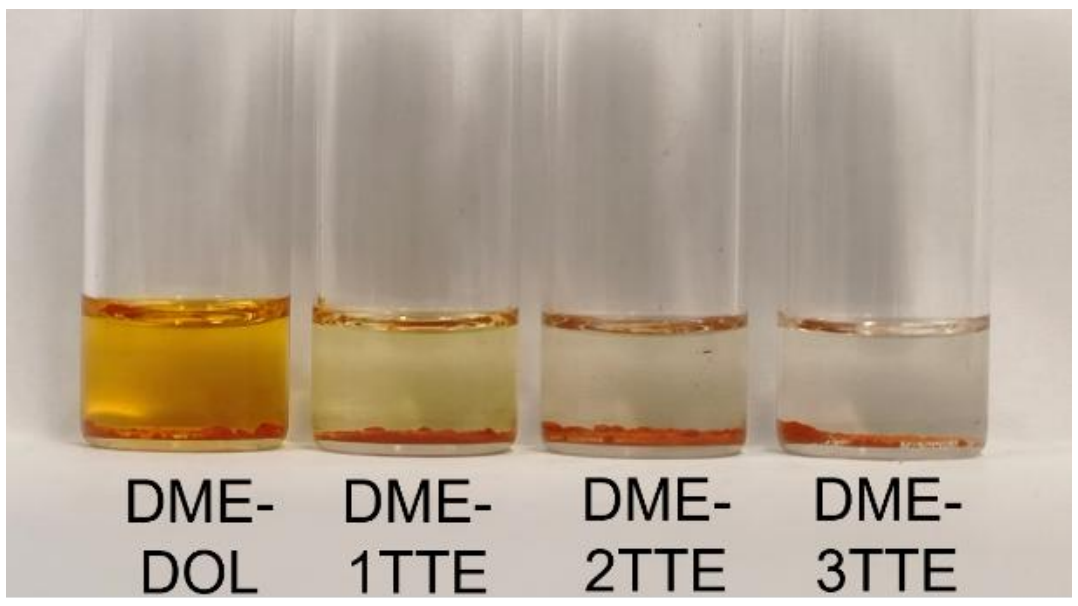

Figure S20. Digital photo of D·I_2 in different solvents after rest for 26 h.

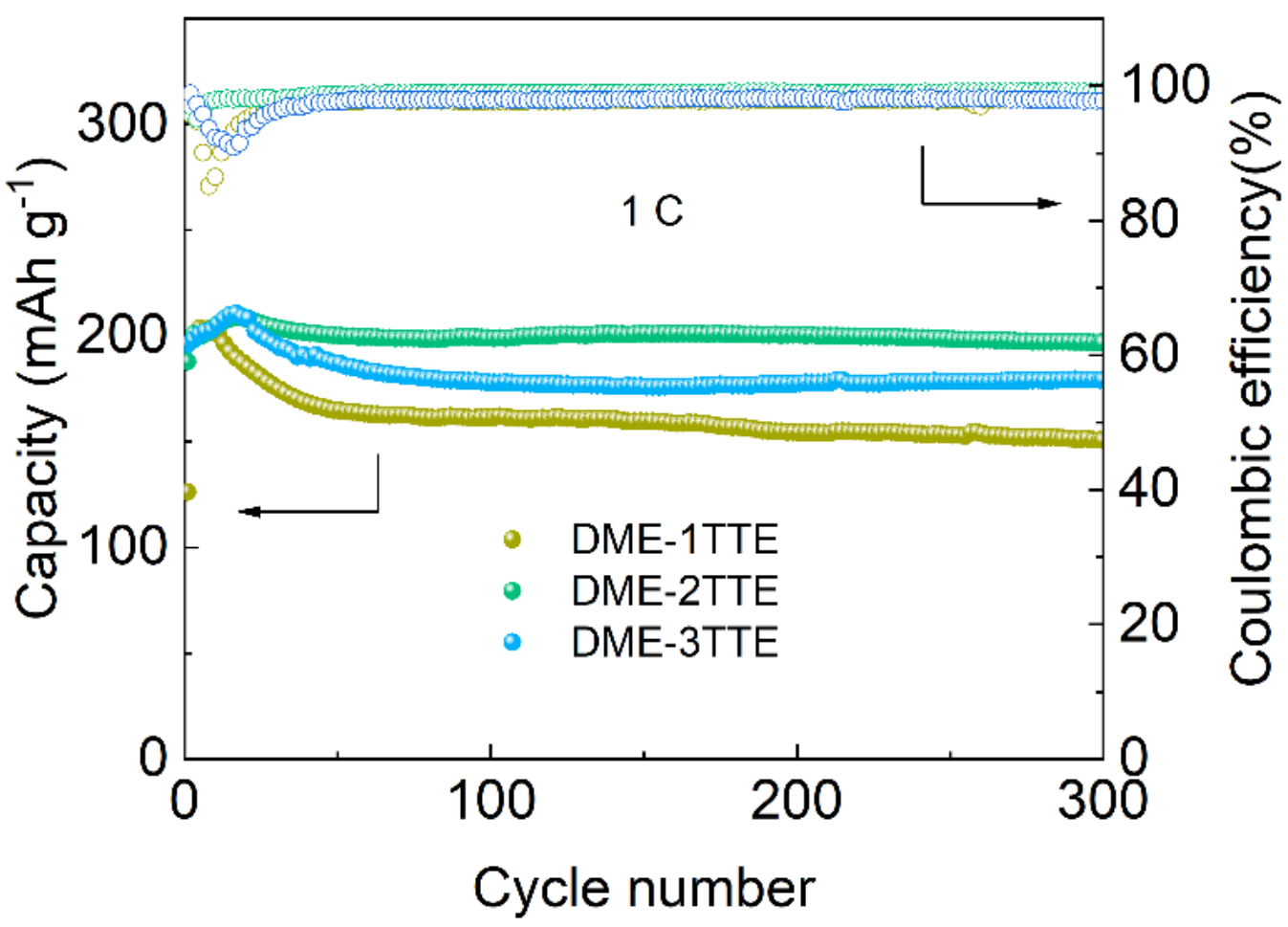

Figure S21. Cycling performance of D·I_2 cathode in DME-xTTE electrolytes.

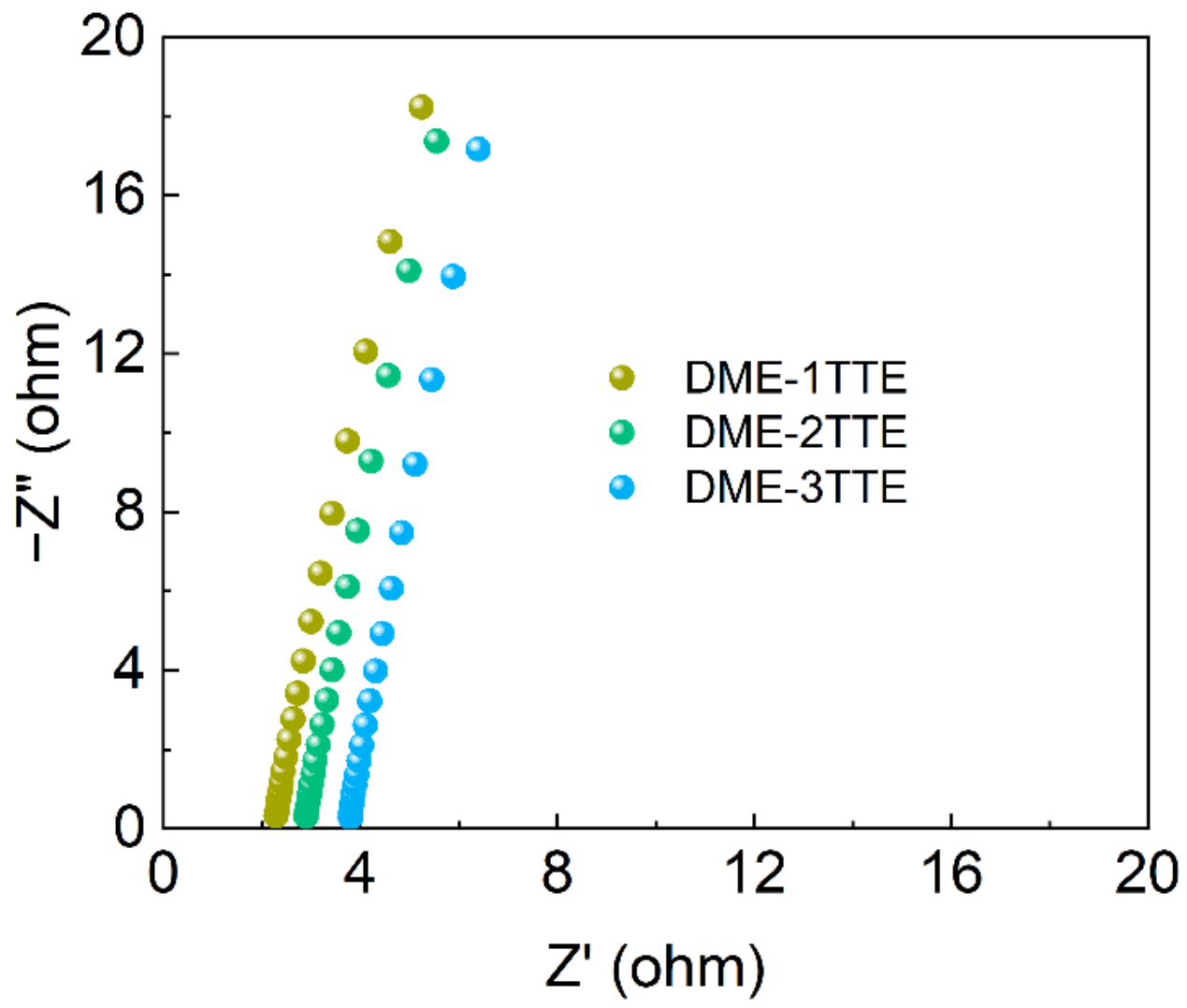

Figure S22. EIS spectra of DME-*x*TTE electrolytes.

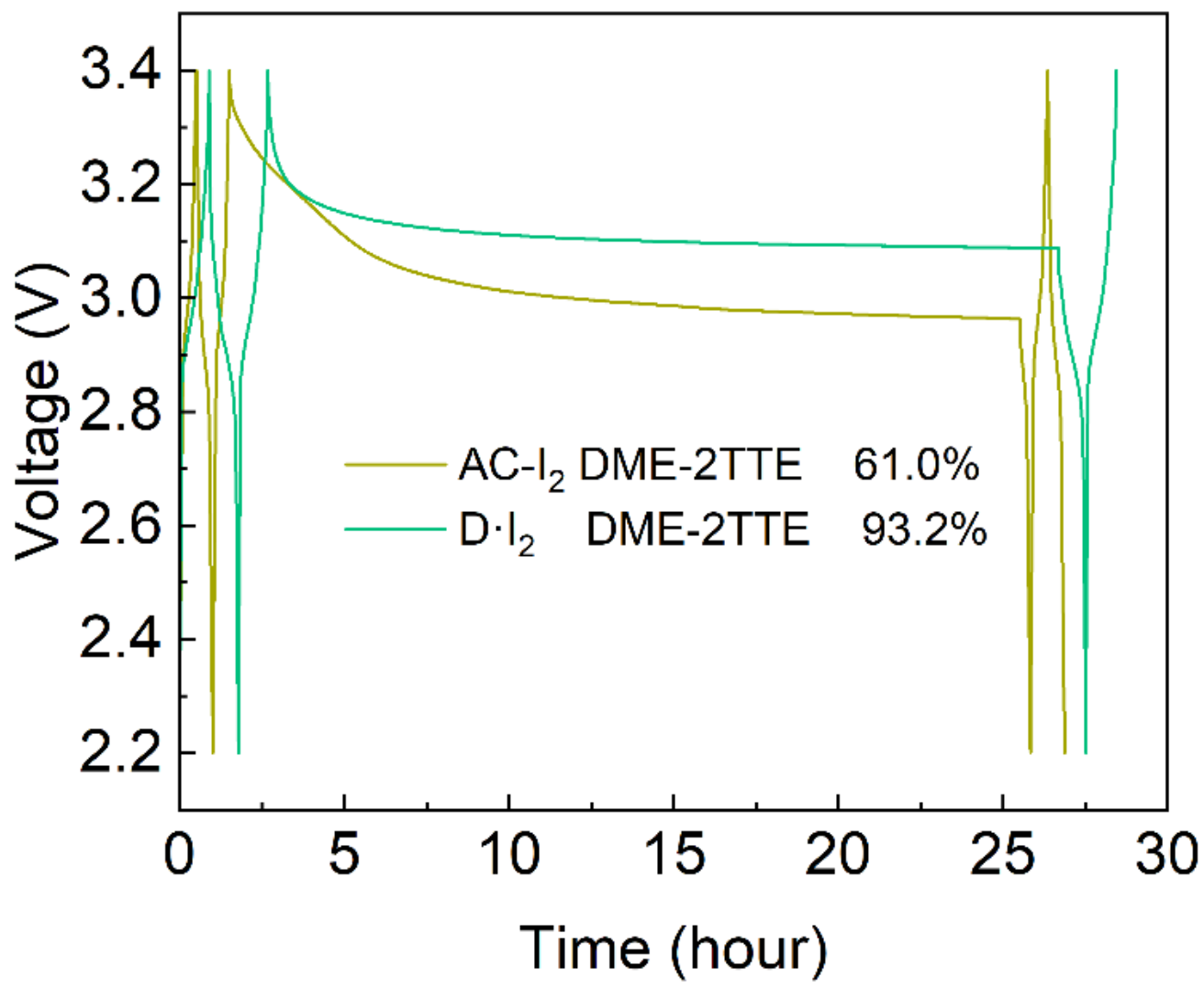

Figure S23. Self-discharge curves. The cells were cycled at 1 C for 20 cycles before the test.

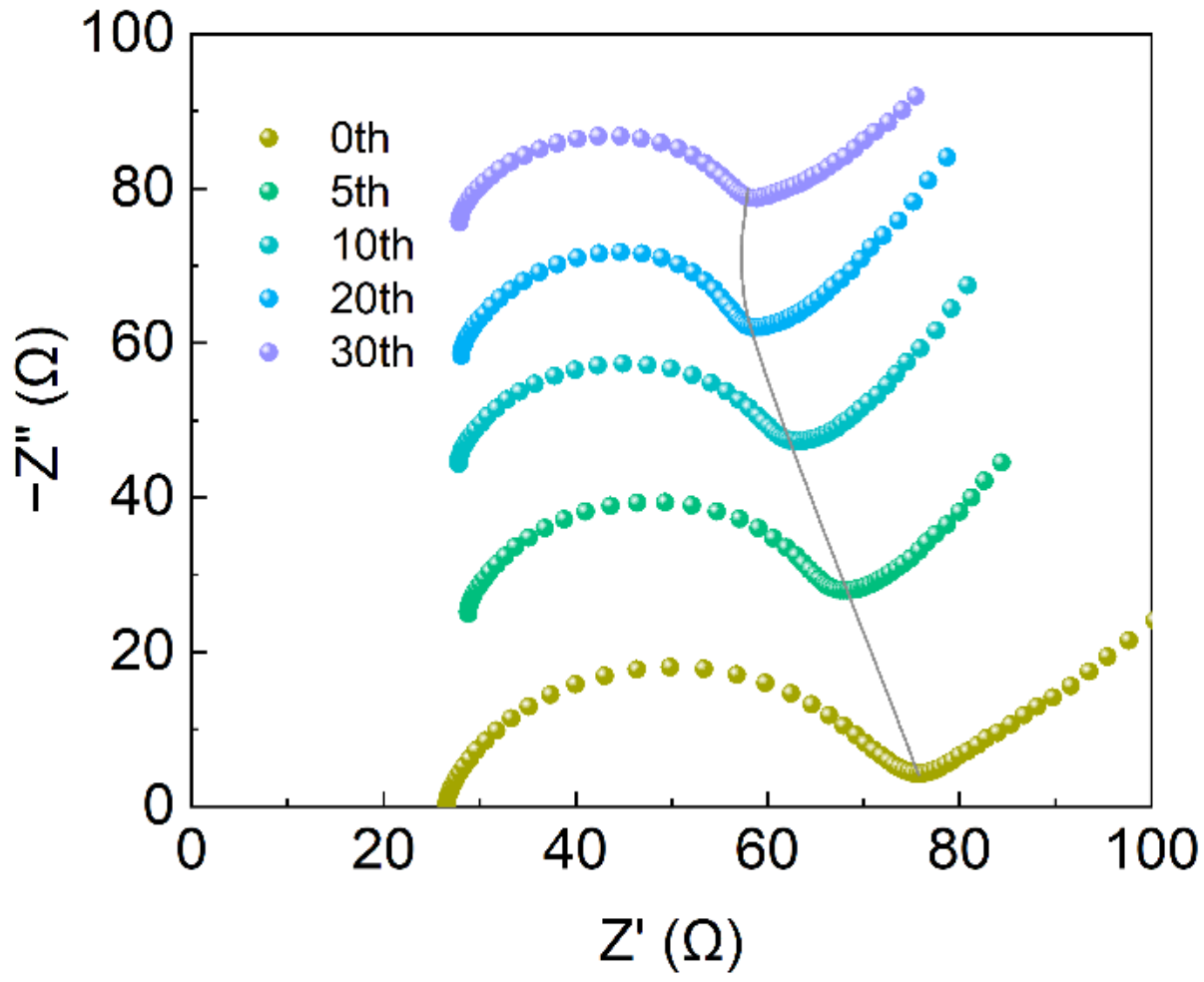

Figure S24. In situ EIS spectra of D·I_2 cathode in DME-2TTE electrolyte.

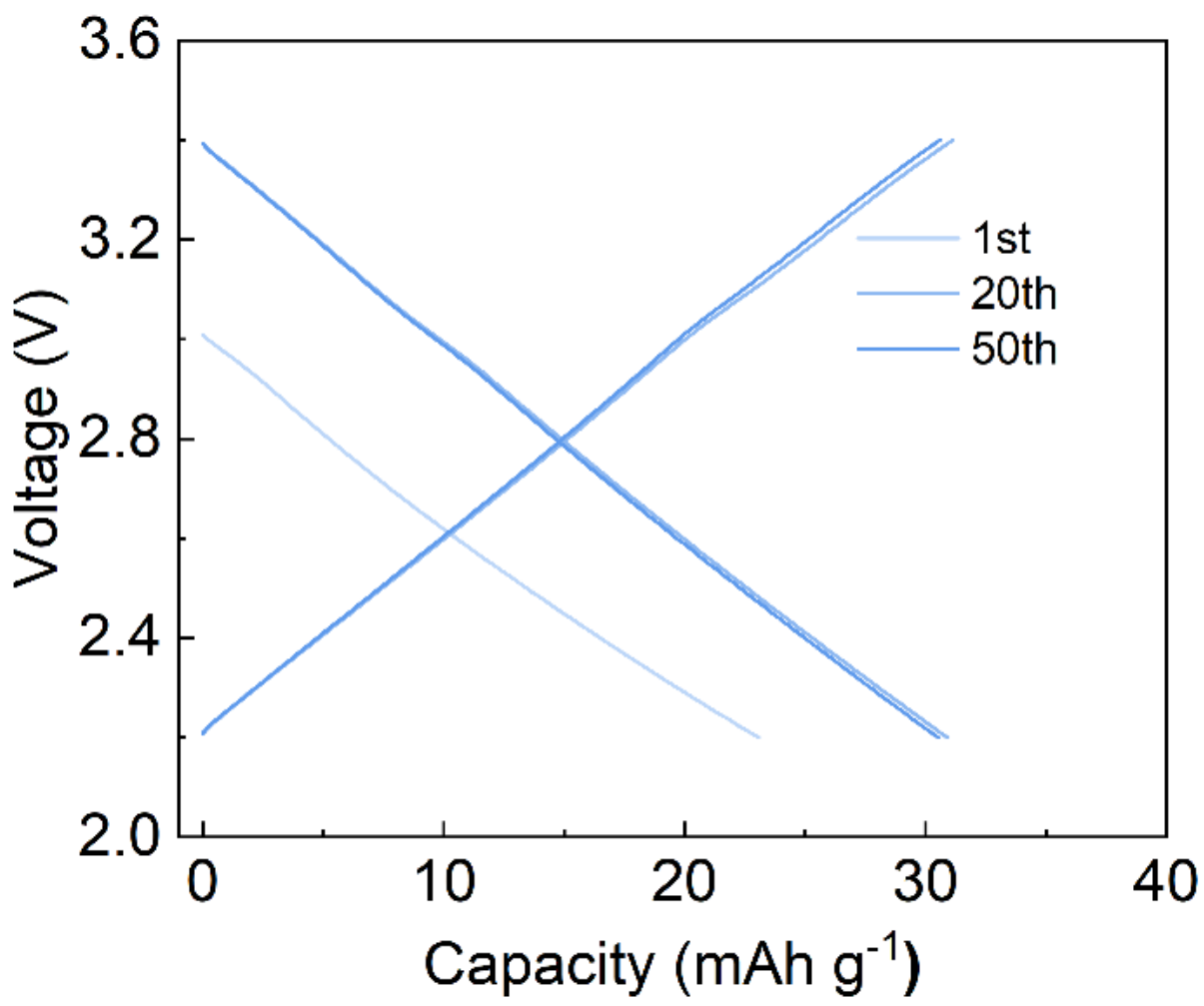

Figure S25. GCD curves of neat carbon electrode (iodine free) at 0.1 C.

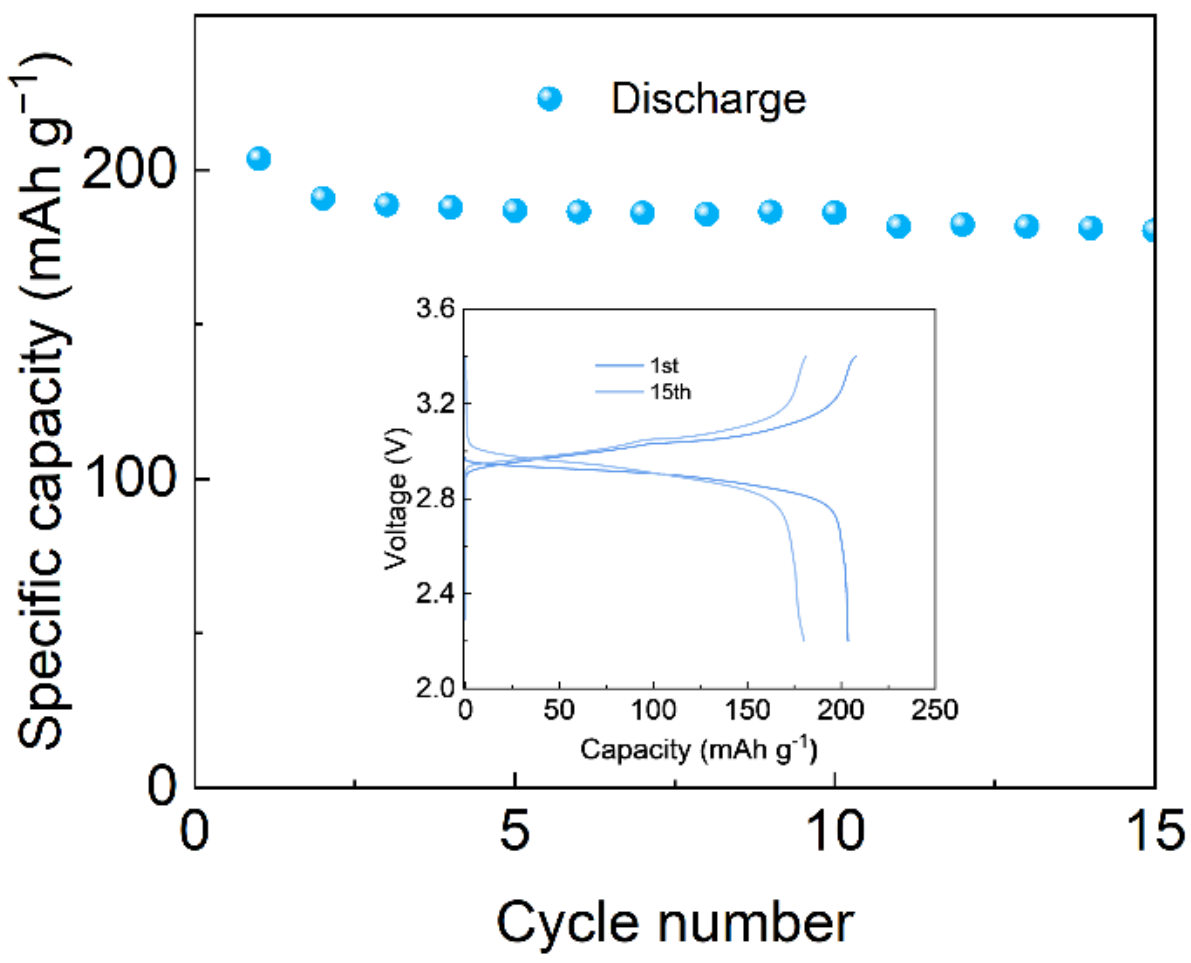

Figure S26. Battery performance of high-iodine-content electrode (90 wt.% D·I_2 +10 wt.% PTFE) under DME-2TTE electrolyte.

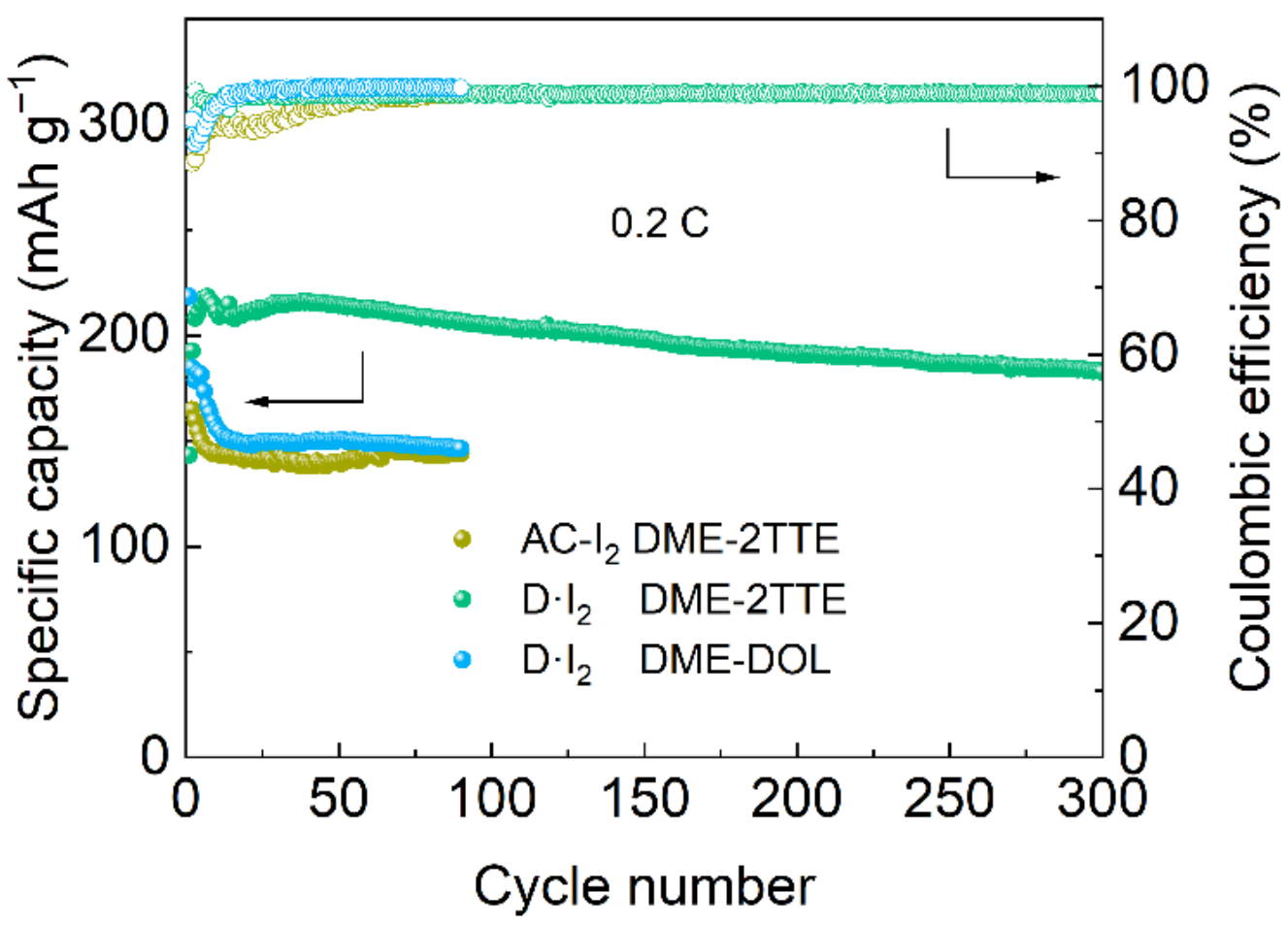

Figure S27. Cycling performance at 0.2 C.

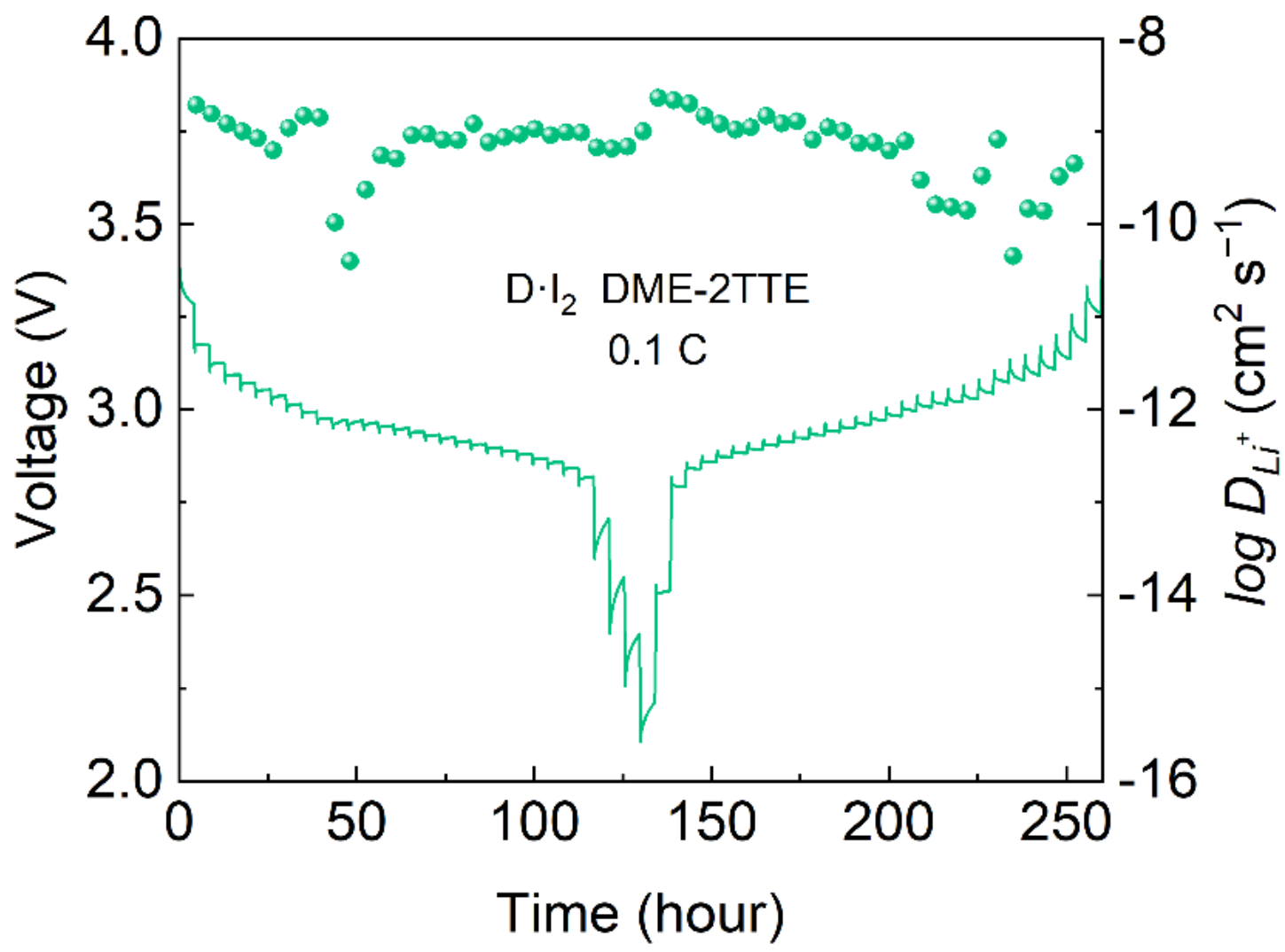

Figure S28. GITT results of D·I₂ cathode in DME-2TTE electrolyte.

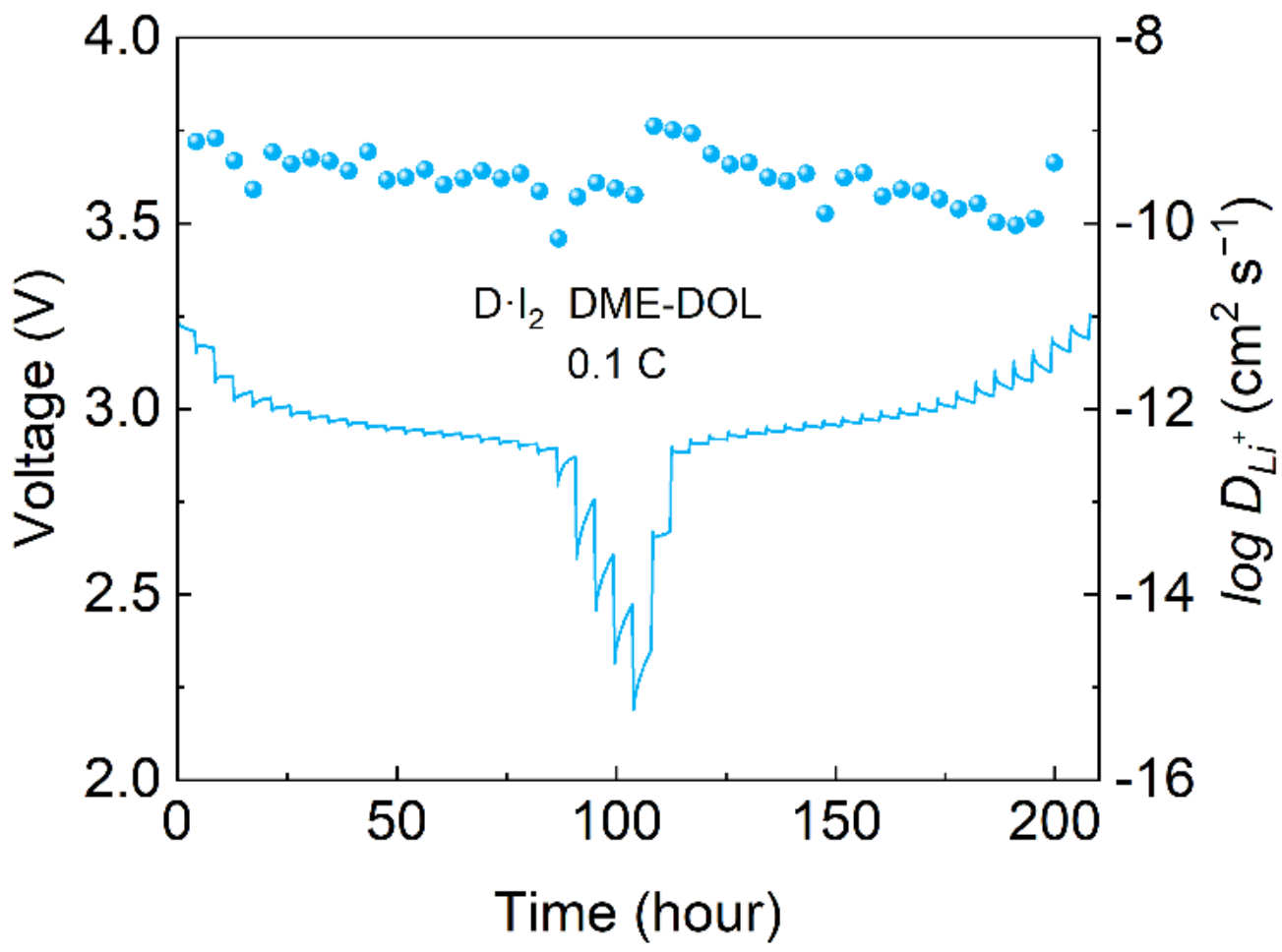

Figure S29. GITT results of D·I_2 cathode in DME-DOL electrolyte.

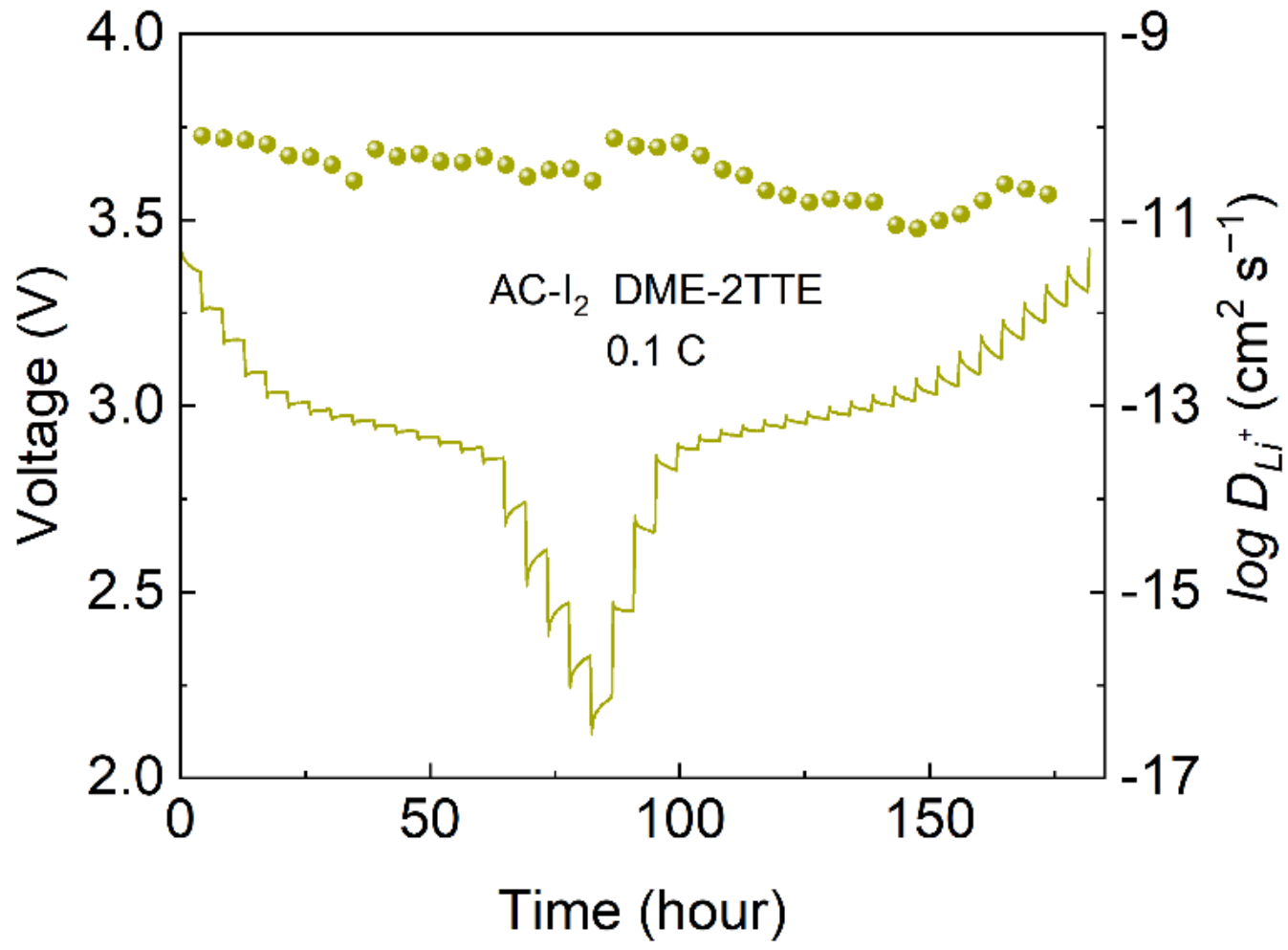

Figure S30. GITT results of AC- I_2 cathode in DME-2TTE electrolyte.

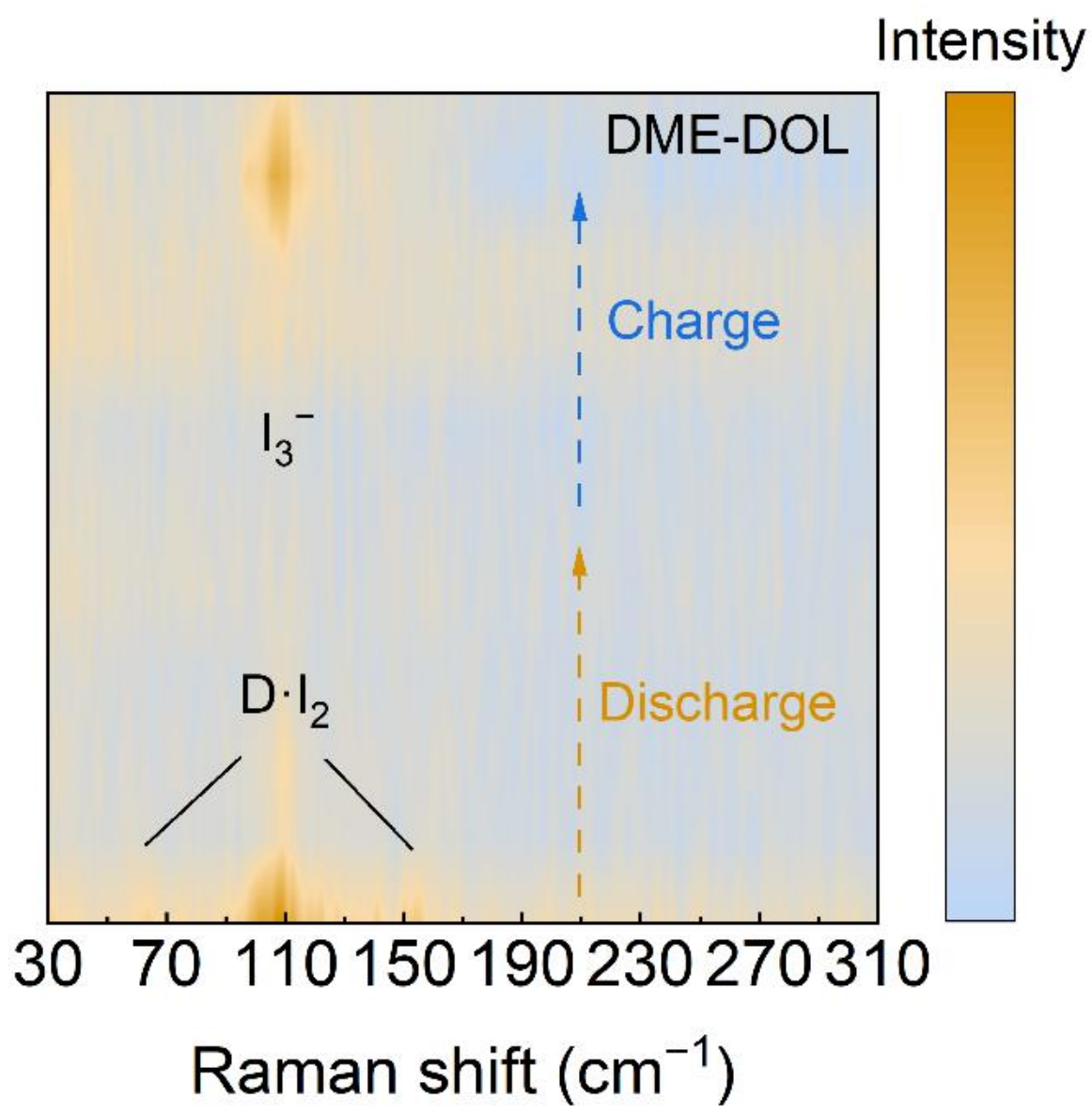

Figure S31. In situ Raman spectra of D·I_2 cathode in DME-DOL electrolyte.

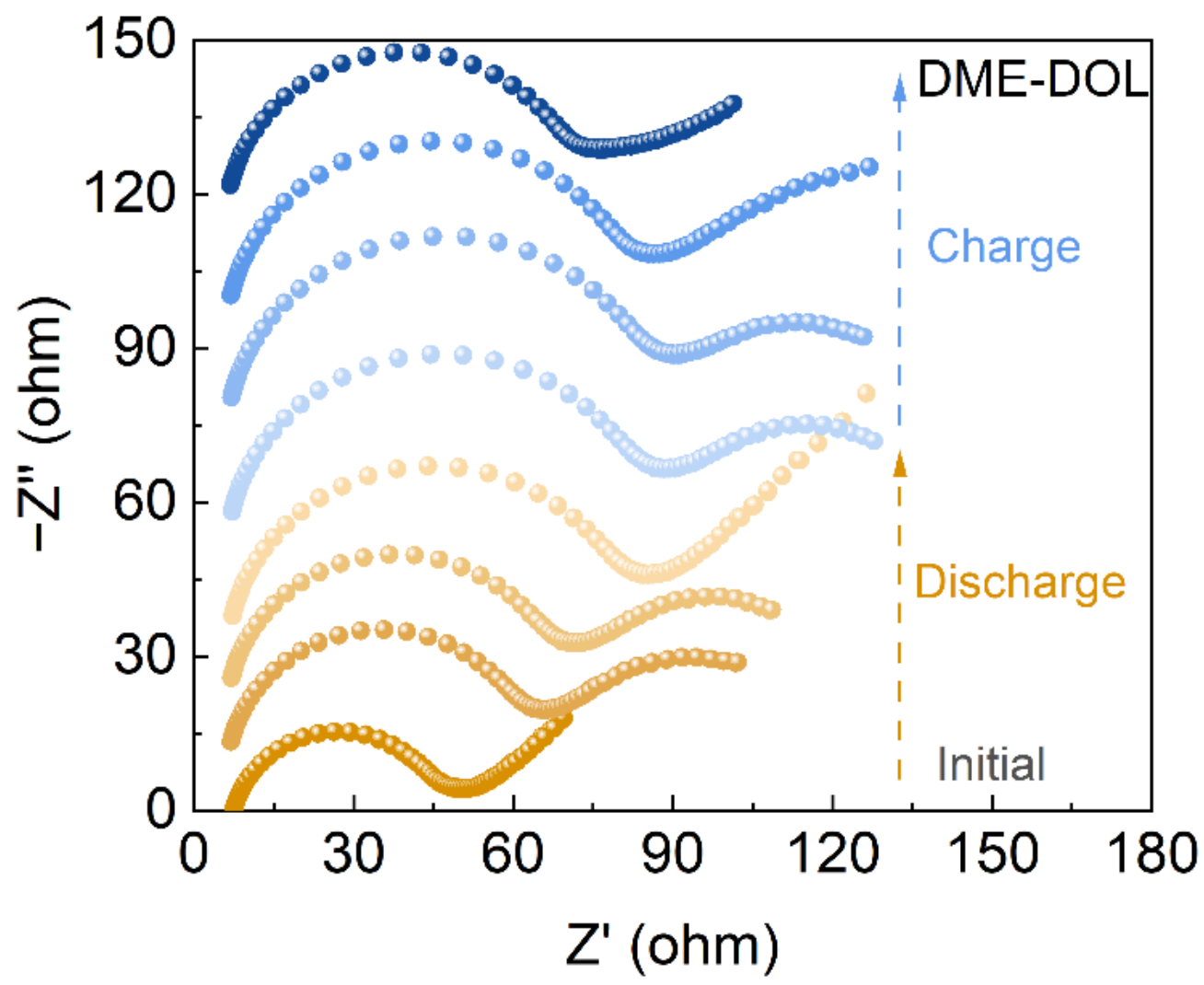

Figure S32. Impedance evolution of D·I_2 cathode in DME-DOL electrolyte.

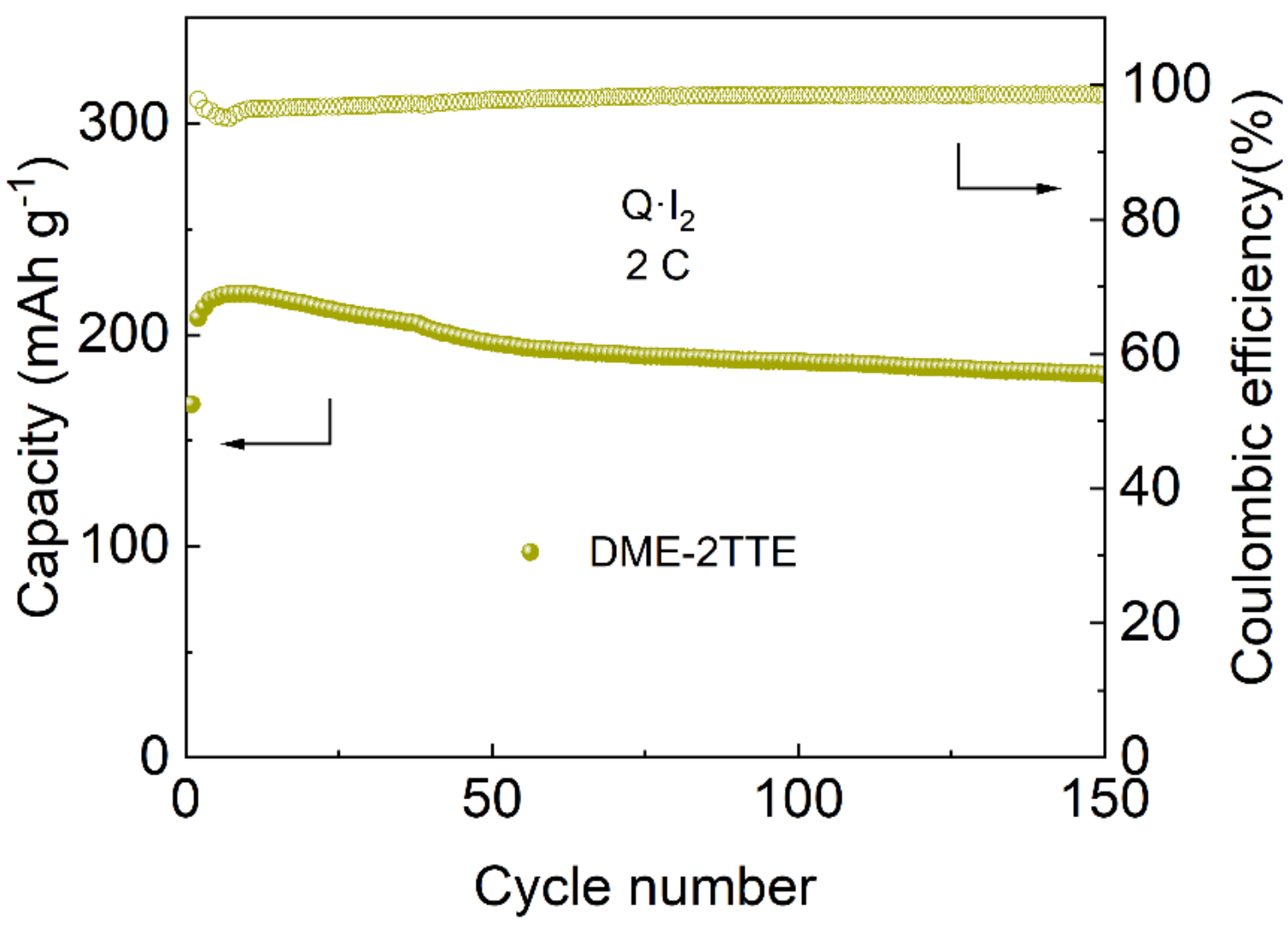

Figure S33. Cycling performance of Q·I_2 cathode in DME-2TTE electrolyte.

Supplementary Tables

Table S1. Theoretical calculation results of iodine complexes.

(At a 1:1 molar ratio of iodine to ligand unless specified)

Iodine with Ligands	Binding energy (eV)	Transferred charge (electron)	I−I bond length (Å)
CH	-0.145	0.005	2.691
DOL	-0.256	0.048	2.718
DME	-0.351	0.06	2.725
THF	-0.395	0.072	2.730
TEP	-0.513	0.077	2.732
DMSO	-0.418	0.097	2.738
Py	-0.538	0.160	2.771
HMPA	-0.591	0.114	2.749
TEA	-0.700	0.217	2.800
DABCO (2:1)	-0.690	0.161	2.779
Quin	-0.79	0.212	2.805
TTE	-0.139	0.012	2.696
DEC	-0.301	0.036	2.715
TFEC	-0.226	0.009	2.705

Table S2. Cycling performance comparison with latest works.

Optimization strategy	Current density (1C=211 mA g^{-1})	Iodine in cathode (wt.)	Performance (mAh g^{-1})	Cycling time	Ref.
PNC@TiN_I_2	23.7 C (5 A g^{-1})	36.1%	136	~3600 h 66000 cycles	Angew, 2026 [6]
Carbonized PPh3@Co-ZIF-8/I_2	23.7 C (5 A g^{-1})	28.56%	120	~2400 h 50000 cycles	Adv. Mater, 2025 [7]
Zn_1Co ACCs/I_2	9.5 C (2 A g^{-1})	25.0%	175	~2600 h 15000 cycles	Energy. Environ Sci, 2026 [8]
AZPY·$2I_2$	34.5 C (8 A g^{-1})	21.9%	160	~5900 h 150000 cycles	JACS, 2025 [9]
Fc-KB-I_2	1 C	63.0%	160	420 cycles	Nat Chem, 2026 [10]
PNZ·I_2	14.2 C (3 A g^{-1})	34.8 %	~185	~560 h 4500 cycles	JACS, 2025 [11]
2D Mxene/I_2 & suspension electrolyte (with n-butanol)	23.7 C (5 A g^{-1})	Nil	300	~3600 h 30000 cycles	Nat. Commun, 2024 [12]
Polymelamine@I_2	71.1 C (15 A g^{-1})	Nil	330	~900 h 20000 cycles	Adv. Mater, 2026 [13]
R-ANF-MOF in DME/DOL (quasi-solid-state electrolyte)	5 C<br>10 C	56.0%	175<br>95.6	450 h<br>3000 h	Angew, 2025 [14]
Methylammonium iodide cathode	5 C	56.0%	217	~4000 h 10000 cycles	Adv. Energy Mater, 2022 [15]
Mo-rGO@PP interlayer	5 C<br>10 C	24.0%	200<br>175	~1350 h 3500 cycles<br>~1000 h 6000 cycles	Adv. Funct. Mater, 2023 [16]
HOF@$Ti_3C_2T_x$-I_2	0.2 C	31.0%	275	~3900 h 300 cycles	Angew, 2023 [17]

D·I_2 cathode & DME/TTE electrolyte	0.1 C	40.0%	215	4000 h	**This work**

Note: Performances are calculated based on average capacity. References in red correspond to Zn−I_2 battery systems, whereas those in blue correspond to Li−I_2 battery systems. **Low-current cycling is more challenging because of exacerbated parasitic reactions.**

References

1. Z. Wang, Y. Wang, B. Py, et al., "DRTtools: Freely Accessible Distribution of Relaxation Times Analysis for Electrochemical Impedance Spectroscopy," *ACS Electrochemistry* 1, no. 12 (2025): 2680-2689, https://doi.org/10.1021/acselectrochem.5c00334.

2. T. H. Wan, M. Saccoccio, C. Chen, F. Ciucci, "Influence of the Discretization Methods on the Distribution of Relaxation Times Deconvolution: Implementing Radial Basis Functions with DRTtools," *Electrochimica Acta* 184, no. (2015): 483-499, https://doi.org/10.1016/j.electacta.2015.09.097.

3. *Gaussian 16 Rev. A.03*; Wallingford, CT, 2016.

4. F. Weigend, R. Ahlrichs, "Balanced Basis Sets of Split Valence, Triple Zeta Valence and Quadruple Zeta Valence Quality for H to Rn: Design and Assessment of Accuracy," *Physical Chemistry Chemical Physics* 7, no. 18 (2005): 3297-3305, https://doi.org/10.1039/b508541a.

5. S. Grimme, S. Ehrlich, L. Goerigk, "Effect of the Damping Function in Dispersion Corrected Density Functional Theory," *Journal of Computational Chemistry* 32, no. 7 (2011): 1456-1465, https://doi.org/10.1002/jcc.21759.

6. Y. Fu, J. Zhong, S. Zhang, et al., "Interfacial Electronic Nanoarchitectonics for Sustainable Zn–I_2 Batteries," *Angewandte Chemie International Edition* 65, no. 13 (2026): e22065, https://doi.org/10.1002/anie.202522065.

7. X. Guo, H. Xu, Z. Qiu, et al., "Heteroatom-Modulated Asymmetric Cobalt Single-Atom Catalysts on MOF-Derived Carbon Enabling Durable Zinc-Iodine Batteries," *Advanced Materials* 37, no. 45 (2025): e14035, https://doi.org/10.1002/adma.202514035.

8. M. Chen, H. Li, H. Xu, et al., "Redirecting Iodine Reduction Pathways by Decoupling Adsorption Energies for Long-Life Zn–I_2 Batteries," *Energy & Environmental Science* 19, no. 3 (2026): 896-905, https://doi.org/10.1039/d5ee06963g.

9. P. Yang, J. Guo, S. Lin, Y. Fu,W. Guo, "Dissolution Inhibition Via Intramolecular N...I Halogen Bond Enables High-Loading Zn-Organic Battery," *Journal of the American Chemical Society* 147, no. 34 (2025): 30820-30828, https://doi.org/10.1021/jacs.5c07277.

10. S. J. Zhang, J. Hao, H. Wu, et al., "Electroactive Ferrocene/Ferrocenium Redox Coupling for Shuttle-Free Aqueous Zinc-Iodine Pouch Cells," *Nature Chemistry* 18, no. 2 (2026): 266-274, https://doi.org/10.1038/s41557-025-01986-7.

11. J. Guo, Z. Zhang, F. Zhu, et al., "Aqueous Zinc-Organoiodine Battery with High Kinetics and Dense Cathodes," *Journal of the American Chemical Society* 147, no. 43 (2025): 39652-39661, https://doi.org/10.1021/jacs.5c13131.

12. W. Yan, Y. Liu, J. Qiu, et al., "A Tripartite Synergistic Optimization Strategy for Zinc-Iodine Batteries," *Nature Communications* 15, no. 1 (2024): 9702, https://doi.org/10.1038/s41467-024-53800-6.

13. Y. Wang, Y. Lv, S. Wei, et al., "Polymerized Melamine Catalyzes Direct I^-/I_2 Conversion Via —N═N— Motif for High Capacity Zn-I_2 Batteries," *Advanced Materials* 38, no. 4 (2026): e15000, https://doi.org/10.1002/adma.202515000.

14. J. Gu, C. Dong, Y. Zhu, et al., "Constructing Matching Interfaces by Amorphous Engineering for Enhanced Lithium Ion Transport in Quasi-Solid-State Lithium-Iodine Batteries," *Angewandte Chemie International Edition* 64, no. 29 (2025): e202507184, https://doi.org/10.1002/anie.202507184.

15. P. Li, X. Li, Y. Guo, et al., "Highly Thermally/Electrochemically Stable I^-/I_3^- Bonded Organic Salts with High I Content for Long‐Life $Li–I_2$ Batteries," *Advanced Energy Materials* 12, no. 15 (2022): 2103648, https://doi.org/10.1002/aenm.202103648.
16. Q. Chen, S. Chen, J. Ma, S. Ding,J. Zhang, "Separation Effect of Graphene Sheets with N, O Codoping and Molybdenum Clusters for Reversible Lithium–Iodine Batteries," *Advanced Functional Materials* 34, no. 2 (2023): 2308272, https://doi.org/https://doi.org/10.1002/adfm.202308272.
17. C. Guo, B. Han, W. Sun, et al., "Hydrogen-Bonded Organic Framework for High-Performance Lithium/Sodium-Iodine Organic Batteries," *Angewandte Chemie International Edition* 61, no. 48 (2022): e202213276, https://doi.org/10.1002/anie.202213276.
